# Natural Biomaterials for Cardiac Tissue Engineering: A Highly Biocompatible Solution

**DOI:** 10.3389/fcvm.2020.554597

**Published:** 2020-10-23

**Authors:** Qasim A. Majid, Annabelle T. R. Fricker, David A. Gregory, Natalia Davidenko, Olivia Hernandez Cruz, Richard J. Jabbour, Thomas J. Owen, Pooja Basnett, Barbara Lukasiewicz, Molly Stevens, Serena Best, Ruth Cameron, Sanjay Sinha, Sian E. Harding, Ipsita Roy

**Affiliations:** ^1^Faculty of Medicine, National Heart and Lung Institute, Imperial College London, London, United Kingdom; ^2^Department of Material Science and Engineering, Faculty of Engineering, University of Sheffield, Sheffield, United Kingdom; ^3^Department of Materials Science and Metallurgy, Cambridge Centre for Medical Materials, University of Cambridge, Cambridge, United Kingdom; ^4^Department of Bioengineering, Department of Materials, IBME, Faculty of Engineering, Imperial College London, United Kingdom; ^5^Applied Biotechnology Research Group, School of Life Sciences, College of Liberal Arts and Sciences, University of Westminster, London, United Kingdom; ^6^Wellcome-MRC Cambridge Stem Cell Institute, University of Cambridge, Cambridge, United Kingdom

**Keywords:** cardiac tissue engineering, natural biomaterial, engineered heart tissue, alginate, silk, polyhydroxyalkanoate, collagen, fibrinogen

## Abstract

Cardiovascular diseases (CVD) constitute a major fraction of the current major global diseases and lead to about 30% of the deaths, i.e., 17.9 million deaths per year. CVD include coronary artery disease (CAD), myocardial infarction (MI), arrhythmias, heart failure, heart valve diseases, congenital heart disease, and cardiomyopathy. Cardiac Tissue Engineering (CTE) aims to address these conditions, the overall goal being the efficient regeneration of diseased cardiac tissue using an ideal combination of biomaterials and cells. Various cells have thus far been utilized in pre-clinical studies for CTE. These include adult stem cell populations (mesenchymal stem cells) and pluripotent stem cells (including autologous human induced pluripotent stem cells or allogenic human embryonic stem cells) with the latter undergoing differentiation to form functional cardiac cells. The ideal biomaterial for cardiac tissue engineering needs to have suitable material properties with the ability to support efficient attachment, growth, and differentiation of the cardiac cells, leading to the formation of functional cardiac tissue. In this review, we have focused on the use of biomaterials of natural origin for CTE. Natural biomaterials are generally known to be highly biocompatible and in addition are sustainable in nature. We have focused on those that have been widely explored in CTE and describe the original work and the current state of art. These include fibrinogen (in the context of Engineered Heart Tissue, EHT), collagen, alginate, silk, and Polyhydroxyalkanoates (PHAs). Amongst these, fibrinogen, collagen, alginate, and silk are isolated from natural sources whereas PHAs are produced via bacterial fermentation. Overall, these biomaterials have proven to be highly promising, displaying robust biocompatibility and, when combined with cells, an ability to enhance post-MI cardiac function in pre-clinical models. As such, CTE has great potential for future clinical solutions and hence can lead to a considerable reduction in mortality rates due to CVD.

## Natural Polymer Based Engineered Heart Tissue

Increasing clinical demands have led to myocardial tissue engineering becoming a prime focus of investigation within the field of regenerative medicine. This novel approach aims to provide a viable alternative and improvement to the traditional pharmacological and interventional therapies, currently available in cardiac medicine, and also to relatively new cell-based techniques such as *in situ* cellular cardiomyoplasty (1–3). The general strategy for cardiac tissue engineering is to combine functional cardiomyocytes and biomaterials with carefully designated characteristics to repair and restore diseased heart tissue (2–5). The selection of these biomaterials is a challenging task due to the strict requirements imposed on the heart TE substrates (2, 3, 6), which are required not only to support cell attachment and alignment, but also to transmit load, provide physiologically relevant stiffness, and be degraded and replaced over time by extracellular matrix (ECM) proteins secreted by cells. Ideally, the myocardial scaffold should allow cardiomyocytes to develop a mature contractile phenotype, and to communicate with adjacent cells. In the native heart tissue, the ECM provides this crucial physiological environment for maintaining the vital functions of cardiac cells. It is logical to assume that the most effective scaffolding materials will be those which possess biochemical composition, structure, and function similar to that of the native cardiac ECM.

This review aims to provide an overview of several naturally occurring biomaterials with particular interest in their synthesis, examples of their use in a range of CTE applications as well as the advantages and disadvantages of each biomaterial assessed. To this end, fibrinogen (through its application in Engineered Heart Tissue) has been explored extensively for the maturation of CMs *in vitro*, disease modeling, and drug screening in addition CTE applications. Furthermore, the adaptation of collagen and alginate to generate biomaterials with properties conducive to CTE are discussed in addition to the use of alginate for the delivery of delivery of factors and drugs that can facilitate cardiac regeneration. Silk and polyhydroxyalkanoates, a family of naturally occurring biomaterials produced via bacterial fermentation, are also explored with particular attention paid to the use of the latter for left ventricular cardiac patches and cardiac valve replacement.

### Fibrinogen and Engineered Heart Tissue

The development of Engineered Heart Tissue (EHT) was pioneered by Thomas Eschenhagen (7) and was created by combining cardiomyocytes and or non-cardiomyocytes within an ECM to form a 3D construct. Such ECM-like gel-based cardiac patches possess the advantage of being easily shaped or cast to the complex geometry of the myocardium, so providing efficient bonding to the native tissue. This platform has developed considerably in the last twenty years, going through an evolution from early constructs utilizing glass tubes with Velcro, to a medium-throughput method using silicone posts and a fibrin extracellular matrix (8). EHTs are now being used as tools for drug screening, disease modeling, and in cardiac regeneration to replace lost myocytes post-myocardial infarction, and are on the cusp of being approved for clinical trials (9).

### Evolution

**Figure 1:**
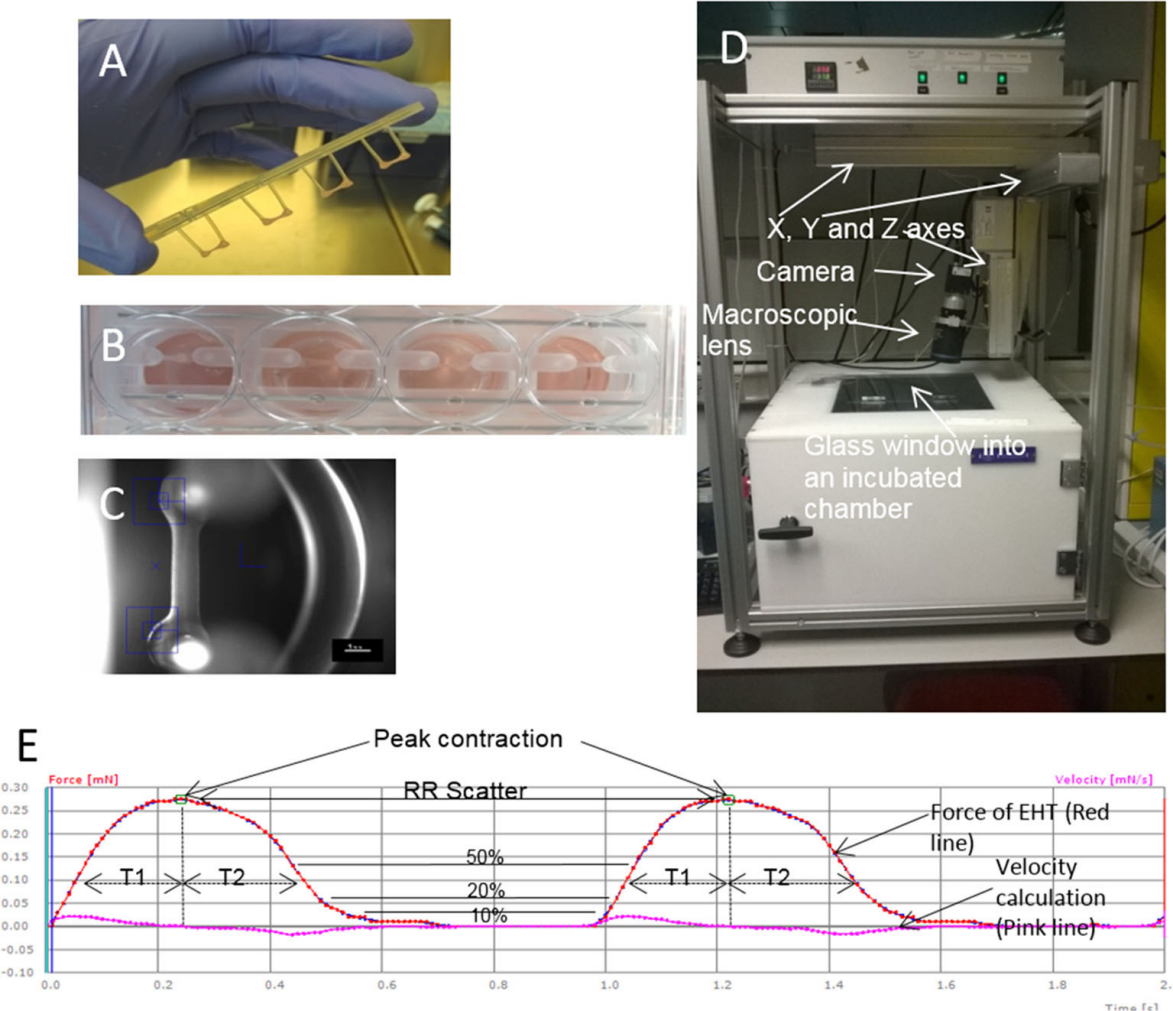
EHTs and the white box. **(A)** Four EHTs attached to a silicone rack are shown, and **(B)** inside media in a 24 well-plate. **(C)** A contraction is recorded by the movement of the blue boxes which pick up the contrast difference between the EHT and the background. **(D)** A picture of the outside of the white box. **(E)** Contraction measurements on traces from the white box. An example of an EHT contracting for 2 s is shown indicating how different parameters are calculated from contractions. Peak contraction is taken at the green boxes and RR scatter as seconds is calculated as time between the two boxes. Time to contraction (T1) is calculated at 10, 20, and 50% of the peak from the midline to the edge of the curve, and relaxation time (T2) is calculated in the same way. Contraction velocity and relaxation velocity are calculated as the derivative of the curve and shown by the pink line. Each small box on the Red and Pink lines shows a frame taken by the white box camera which runs at 100 f.p.s.

The first report of EHT, *in vitro*, used isolated embryonic chick cardiomyocytes mixed with collagen to form a contracting 3D construct, resembling the *in vivo* heart tissue (7). After culturing *in vitro*, the cardiomyocytes produced a spontaneously and coherently contracting 3D matrix with a highly organized myocardium-like structure and typical functions of myocardial tissue. This seminal piece of work by Eschenhagen reported that an increased force was generated like in *in vivo* heart tissue including: increasing extracellular Ca^2+^; a positive force frequency relationship; and a positive Frank-Starling mechanism (7). Later, the same group reported the long-term survival of neonatal rat cardiomyocytes in the scaffold obtained by a similar gelation step of the collagen solution (10). This artificial heart tissue showed an increase in beating power up to 18 days of culture *in vitro* with a maximal contraction force of 2–4 mN. The model was then developed by making circular EHTs using neonatal rat heart cells combined with collagen I and Matrigel which resulted in more mature cells, better myofiber alignment, coupling, and contraction force (11), and then developed further by employing gene transfer (12). These modifications significantly improved the force of contraction of the resultant gel. After 12 days in culture, the blended Matrigel-collagen construct was implanted into infarcted rat hearts. A well-organized and vascularized heart muscle structure developed after 14 days of implantation (13). Moreover, this implant provided significant improvement to the cardiac function in terms of attenuation of further myocardial dilation and increase in the wall thickness. EHTs are used in tissue regeneration and drug screening approaches and so reproducibility between constructs is essential. Hence, in 2010 the EHT generation process was updated to a medium throughput method using the reaction of fibrinogen and thrombin to create a hydrogel (8). The fibrin hydrogel forms around two silicone posts (Figures 1A,B) which give mechanical load to the constructs in an auxotonic fashion. EHTs beat spontaneously and custom-made software can detect the deflection of the silicone posts and can then produce contraction kinetics automatically (8, 14) (Figures 1C–E). EHT contraction kinetics mature over time *in vitro* and therefore can be used as a surrogate marker of adequate construct performance prior to grafting or drug screening.

### Improvements Over Monolayers

The importance of 3D culturing of cells in an EHT platform has been shown to be superior to conventional 2D monolayer techniques in many studies. For example, isolated cells from 3D EHT have larger catecholamine responses than cells obtained via the standard 2D monolayer techniques (15). However, cell capacitance levels were reported to be smaller than adult cells with both 2D and 3D approaches, demonstrating that adult maturity has yet to be reached (15). 3D EHTs have also been shown to have 1.8-fold larger sodium current density than 2D monolayers, with EHT up-stroke times approaching adult human myocardium levels (16). Tiburcy et al. (17) also investigated 3D vs. 2D culture gene expression and reported a higher level of adult gene expression with 3D EHTs. These results show that culturing cells in a 3D environment using an EHT platform with load can increase multiple parameters associated with cardiomyocyte maturity; however, further maturation strategies are needed to reach adult levels.

### The Need for Maturation Strategies

Any tissue engineering technology must recapitulate the target tissue *in vitro* to enable it to be a reliable model and maximize efficacy for tissue engineering approaches. In a mature EHT, human induced pluripotent stem cell-derived cardiomyocytes (hiPSC-CMs) become aligned and can generate calculable force contractions with certain adult myocardial characteristics, but forces are often relatively weak when compared to native adult heart tissue. A range of strategies have been used to mature EHT cardiomyocytes to make them adult like, including: (1) electrical and/or mechanical stimulation; (2) hormones/growth factors; (3) using different culture techniques; and (4) adding secondary cell types (17–25). The body of work so far has shown that some parameters of heart tissue can be matured, however, a true adult cardiomyocyte phenotype has not been reproduced.

One of the hallmarks of cardiomyocytes is the contraction and force production from these cells. In early work, force of EHTs was measured at 0.3 mN which is much lower than heart muscle (20 mN), however, recent publications have shown improvement on this method. Mechanical load in an auxotonic fashion and insulin addition to the media were shown to have a positive inotropic effect on culturing neonatal rat cardiomyocytes (18). Custom-made bioreactors also help mature tissue constructs with the addition of vitamin C, fibroblasts, and increasing static stress (21). In this publication, stem cell-derived cardiomyocytes were selected out by using an antibiotic purification method and made into constructs from clusters without dissociation. This approach produced forces of 4.4 mN/mm^2^ which is only five-fold lower than the adult myocardium. Adult levels of force have been shown in constructs when comparing force per unit area (24, 26). In these approaches, the width of the construct is thinner, which increases the force per unit area. Force/area is further increased when stretch is applied. Construct remodeling and the reduction in width occurs over time and is thought to be largely accomplished by non-cardiomyocytes. At the time of peak force of contraction, the fibroblast to cardiomyocyte ratio was reported at ~1:1 (similar to the adult myocardium), showing non-cardiomyocyte proliferation since ~30% of the total cell number were fibroblasts at baseline (17). Even though all the studies have reported improvements in maturation parameters to a certain degree, the most adult-like tissue formed is still relatively immature when compared to the adult myocardium in terms of conduction velocity (up to 25.8 cm/s when compared to 60–70 cm/s in adult myocardium) (17, 25). A recent study has reported new adult morphological characteristics not present in current *in-vitro* EHT models. By subjecting early stage iPSC-CMs (day 12 just after beating) to an intense electrical stimulation protocol over 4 weeks, where constructs were stimulated by increases of 0.33 Hz/day from 2 to 6 Hz. Adult tissue ultrastructure including transverse tubules and functional calcium handling were present along with oxidative metabolism and a positive force-frequency relationship. However, the conduction velocity reported (25.0 ± 0.9 cm/s) and force generated was still comparable to other methodologies presently available (25).

### Drug Screening

EHTs can be generated easily with minimal variation and they have similar characteristics to heart tissue which means that they are suitable for drug toxicology (8, 27). Moreover, because EHT can be produced reproducibly and quickly, they can be used to test multiple drugs for contraction abnormalities or cardiotoxic actions (28, 29). Many of the drug responses of iPSC-CM EHT are similar to normal human trabeculae, although, there is still a maturity difference between iPSC-CMs and adult cells (27). Lemoine et al. (16, 30) showed that cells cultured in EHTs were suitable for testing I_Kr_ block using proarrhythmic drugs, and also were not overly arrhythmic to clinically safe compounds. Therefore, iPSC-CM EHT allow for drug toxicology to be carried out with abundant material and could help pharmaceutical companies in lowering the rejection rates of drugs during Phase I clinical trials. EHTs of micro-dimensions based on collagen type I (31) or a mixture of collagen I and Matrigel with human embryonic stem cell-derived cardiomyocytes were also used as cardiac models for preclinical drug screening (32). There has already been encouraging take-up of hPSC-CM as a platform for Pharma and the addition of commercially available engineered heart tissue, from companies such as NOVOHEART or Tara Biosystems, allows the drug companies to access standardized and validated constructs.

### Disease Modeling

Having heart constructs which are similar to heart tissue allows for disease modeling *in-vitro*. Hypertrophic cardiomyopathy affects 1 in 500 of the population and is difficult to model in 2D culture because it is primarily a defect in cardiac contraction. Contraction abnormalities have been shown using EHTs caused by mutations in the Myosin Binding Protein-C (MyBP-C), including shorter relaxation and contraction times (33–35). Moreover, mutated EHTs showed an increased Ca^2+^ sensitivity, as seen in cardiac muscle from patients, and increased sensitivity to verapamil, isoprenaline, and EMD 57033. CRISPR/Cas9 is an exciting technology that can be used in conjunction with pluripotent stem cells and EHTs to generate tissues with patient specific diseases (36). This technology has been taken advantage of in modeling both dilated and hypertrophic cardiomyopathies (DCM, HCM), where point mutations in MYH6, ACTC1, or PRKAG2 cause HCM, and mutations truncating the massive protein titin cause DCM (31, 37–39). These approaches show that point mutations can be modeled accurately in EHTs and the mechanistic insights into the patient-specific disease can be worked out. Taking advantage of the EHT system uses fewer animals while being able to model complex diseases like cardiomyopathies. Recently, hydrogel technology has been taken advantage of to make a chamber resembling a ventricle (40). The ejection fraction of 2% and stroke volume are far less than a ventricle of the same size, however, this marks an important improvement in the field.

### A Tool for Cardiac Regeneration

*In-vivo* cardiac regeneration has always been one of the goals of tissue engineering, because heart failure is characterized by the irreversible death of cardiomyocytes and a persistent 5-year mortality of 50% (41). The current treatments that exist unfortunately are unable to replace the muscle that is lost post-myocardial infarction and instead retard progression of the disease via a variety of other mechanisms. EHT technology could become a novel and viable treatment option to restore lost muscle and aid in contraction of the failing heart (42, 43). EHTs can be fused together to create larger constructs 15 mm in diameter and 1–4 mm in length (18). These larger constructs can be wrapped around rat hearts and have shown improvements in an infarction model. Larger EHTs (5–7 million cells) were developed for a guinea pig model with substantial cryo-injuries: the increased size for guinea-pig relative to rodent was a step closer to human dimensions (44). Unexpectedly, the cells inside the EHTs proliferated to such an extent that the constructs became substantially larger at 28 days. This experiment showed proof of concept in a larger animal model improving left ventricular function, including returning fractional shortening to levels seen before injury. Functional improvement has been shown with EHTs in rats (18, 45), guinea pigs (44), and large pigs (46). A number of mechanisms have been proposed for the positive effect including increased vascularization into the scar area, secretion of paracrine factors, direct support of contraction, reduced fibrosis, activation of the immune system, and reduction of scar size. None have been categorically shown to solely explain the functional effect, however, an interesting paper from Vagnozzi et al. (47) have shown activated macrophages elicit similar responses to directly injected bone marrow mononuclear cells and cardiac progenitor cells.

**Figure 2:**
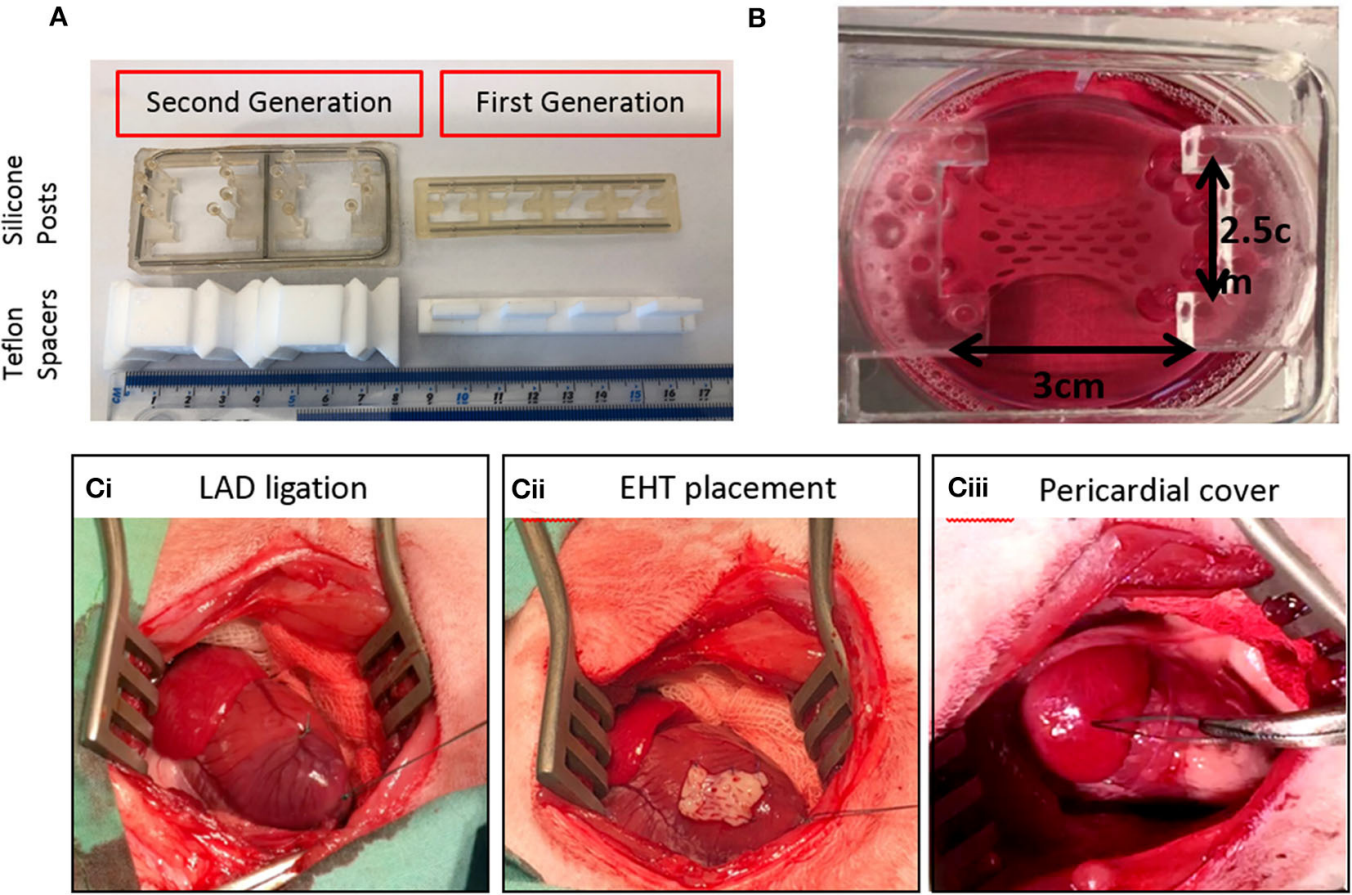
Upscaling of EHTs to six well-format and use in a rabbit myocardial infarction model. **(A)** First generation and second generation EHTs with their Teflon spacers and silicone posts. **(B)** A live upscaled EHT in a six well-plate. **(Ci)** Left Anterior Descending (LAD) coronary artery ligation is shown on a rabbit heart with the ribs held open. **(Cii)** The EHT is attached to the heart with sutures. **(Ciii)** The pericardium is returned over the EHT.

Feasibility and efficacy has also been shown in animal models but clinically relevant EHTs (10 cm × 10 cm) are likely to be necessary for a regenerative medicine approach in heart failure patients because of the large number of cells lost during myocardial infarction (up to one billion) (46, 48, 49). As well as creating larger patches, the generation of a suitable number of cardiomyocytes for human use are needed. There have been dramatic advances in differentiation protocols used recently. For example, using a 3D suspension spinner flask method, cell numbers of the order of 10^9^ have been produced in 1 L flasks (50). Using microcarriers to increase surface area per volume may also enable upscaling (51). Another major hurdle is to maintain viability of the grafts since the typical inter-capillary distance is just 20 μm and clinically relevant grafts would be from millimeters to centimeters in depth. It is likely that vascularization of EHT *in-vitro* will be critical to long-term survival of grafts. Various methods being explored include co-culture with endothelial cells, 3D bioprinting, and microfluidic systems (49, 52–54). Electromechanical integration of the grafts is another hurdle to overcome, since a fibrotic interface is often seen and can reduce the chance of definitive electrical coupling occurring (44). Minimizing the inflammatory response with adequate immunosuppression may reduce fibrosis. Alternatively, research is currently being carried out to create universal donor hiPSC-CM lines which could eventually be used to create hypo-immunogenic patches which are simply prescribed in clinics as an off-the-shelf treatment option for patients with heart failure (49, 55). Finally, the development of pathological ventricular arrhythmia has been a concern in the field; however, this may be related to the mode of delivery since intramyocardial delivery seems to appear more associated with arrhythmia post-grafting (56, 57). Several published studies using epicardial patch placement (e.g., Figure 2) have reassuringly not yet shown any convincing evidence of arrhythmia during the early integration phase, despite evidence of functional improvement vs. controls (46, 48).

Overall, these simple collagen and fibrinogen hydrogel constructs form an excellent substrate to allow stem cell-derived cardiomyocytes to function and mature and have advantages in terms of improved stability and low arrhythmogenicity compared with cell injection only. The ease of reproducibility between laboratories also confirms their robust nature. While their simplicity is a virtue, also for the regulatory process, it does not take advantage of improvements that might be introduced by design of advanced materials or incorporation of other cell types. Other advanced natural materials will now be considered.

## Collagen Modification in Myocardial Tissue Engineering

### Advantages of Collagen for Myocardial TE

In the search for an ECM-mimetic substrate, proteins, and especially ECM-derived biopolymers, have been viewed as potential resources for many heart TE platforms, owing to their intrinsic ability to perform very specific biochemical, mechanical, and structural roles (58, 59). Among them, collagen, with its inherent biocompatibility (superior to that of many other natural polymers), bioactivity [due to the presence of appropriate binding ligands for cardiac cells attachment (60–64)], modifiable biodegradability, and low antigenicity, has emerged as a key material for the development of myocardial 3D biomimetic substrates (6, 60, 61). Collagen scaffolds are also versatile, with many relevant physical, chemical, mechanical, and morphological properties being tailorable to achieve specific functions. For example, by varying fabrication conditions, 3D architecture (percolation diameter, pore size, shape, and alignment) can be controlled to facilitate cell infiltration and nutrient diffusion (65–69), while by changing composition (e.g., by adding other proteins) and crosslinking conditions, scaffold specific functions can be varied to match the properties of the native tissue (70–72). Collagen can also be extracted in large quantities, cheaply and in relatively high purity from a wide range of tissue sources (including skin, tendon, etc.) using a simple acid extraction procedure followed by neutralization (73–76).

### The Collagen Family

**Figure 3:**
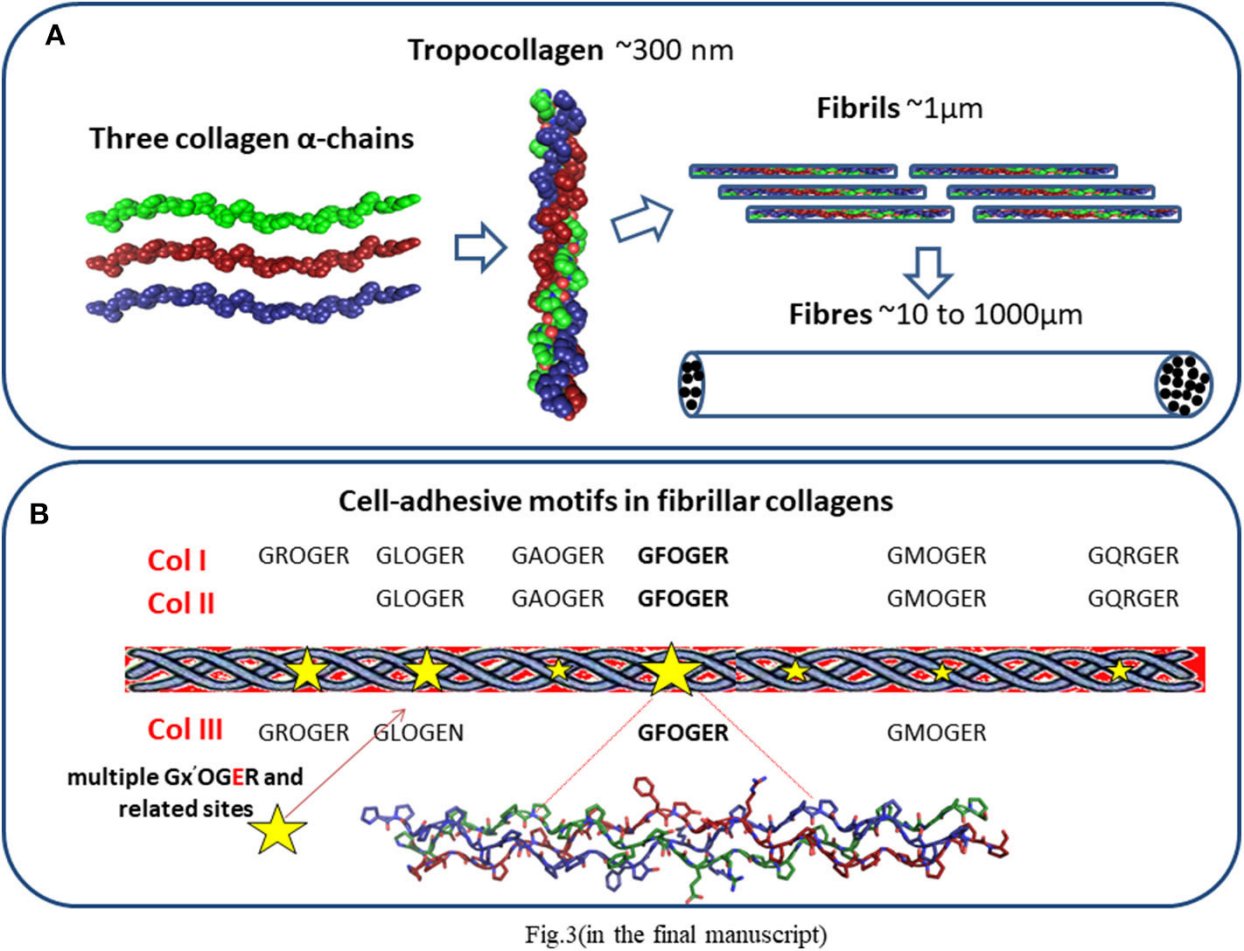
**(A)** Collagen structure. Three polypeptide subunits (α-chains) with a common triple helix configuration. These triple helices comprise a molecule of tropocollagen, the basic building block of collagen fibers and fibrils. **(B)** Distribution of cell-adhesive sequences in fibrillar collagens.

Collagen comprises a family of molecules with a common triple helix configuration of three polypeptide subunits, known as α-chains. These triple helices comprise a molecule of tropocollagen, the basic building block of collagen fibers (Figure 3A). To date, 28 types of collagen have been identified and described in varying detail (62, 63, 77). The best known and the most abundant are fibrillar collagens I, II, and III, each containing triple-helical ligands, GxOGEx′, that support cellular activity mainly through their interaction via cell-associated integrins α1β1, α2β1, α10β1, and α11β1 (62, 63) (Figure 3B). The strength of cellular adhesiveness of each of these integrins is largely governed by the intrinsic affinity of the individual receptor toward a specific collagen ligand. The structural diversity observed across the 28 collagen types is reflected in differences in their cell-adhesive sequences (62, 63). The distribution of these sequences in the fibrillar collagens and their resulting affinities toward supporting integrin ligation have been reported (63). It was established, for example, that the GFOGER motif is the highest affinity ligand for α2β1 and α11β1 receptors while GLOGEN has been identified as a preferred binding sequence for α1β1 and also α10β1 integrins (63, 77, 78). The cells found in the heart include cardiomyocytes, endothelial cells, smooth muscle cells, and fibroblasts. Although endothelial cells (79) are the most prevalent cell type by number, cardiomyocytes constitute more than 70% of the total cardiac tissue volume (80). They express the integrin subunits α1, 3, 5, 6, 7, 9, and 10 which are associated with β1 (81, 82), with α1 and 10 being specifically collagen-binding integrin subunits. Collagen therefore has an abundance of potential ligand sites to promote cellular activity during myocardial tissue regeneration.

### Collagen in Myocardial ECM

In the human body, collagen, in particular fibrillar type I, is the main constituent of the ECM of many hard and soft tissues (2, 6, 60, 61) providing both the structural and biological support to resident cells. Myocardial ECM in particular consists roughly of 75–80% fibrillar collagens, mainly type I (up to 85%) and type III (up to 15%), with up to 5% of type V (2, 83, 84). Synthesized by cardiac fibroblasts, they provide elasticity and structural integrity to cardiac tissue and interact with integrins mediating cellular adhesion (2, 78, 85, 86). Jointly, they support myocyte alignment and contribute to matrix resistance to deformation during the cardiac cycle, playing an important role in the maintenance of myocardium shape, thickness, and stiffness. Based on this knowledge and taking into account that tissue engineering is in essence a technique for imitating the extracellular matrix, it is not surprising that much research effort has been focused on the use of collagen to create bio-mimetic artificial heart tissue (2–4, 60, 61).

### Use of Collagen in Different Cardiac TE Strategies

There are currently two broad strategies within cardiac tissue engineering (74, 75):

1) *in situ* delivery of cells into the infarcted myocardium using injectable gels, and 2) *in vitro* construction of cell-populated 3D scaffolds (in the form of gel or of lyophilized sponges/meshes) that can subsequently either be implanted *in vivo* on the infarcted myocardium or used *in vitro* as artificial cardiac models for biomedical studies and pharmaceutical development.

### *In situ* Injectable Gel Substrates

The efficiency of the delivery of cardiomyocytes via epicardial injection (known as *in situ* cellular cardiomyoplasty) has been improved via the use of an injectable gel. This treatment possesses serious drawbacks, such as, for example, death or migration of up to 90% of implanted cells and lack of mechanical or electrical contacts between the injected and host cells (1, 2, 87). The injectable gel approach (1–3, 87, 88), aims at minimally invasive surgery, and collagen alone or in combination with other natural polymers, such as chitosan (89) and fibrin (90) has been explored as an *in situ* gel-delivery system. However, the use of collagen for this application has been restricted due to insufficient stiffness (20–80 Pa for 1–3 mg/ml of type I collagen) (2), high hydrophilicity and low viscosity (91) of its hydrogels which, in turn, may provide insufficient mechanical support to the diseased myocardium. Recent advances in this field include biohybrid hydrogels based on collagen and other polymeric molecules with and without bioconductive properties (92–94). For example, in 2015, Xu et al. (94) reported the efficiency of hybrid hydrogels of thiolated collagen with multiple acrylate containing oligo copolymers for myocardial regeneration. These hydrogels were populated with bone marrow mesenchymal stem cells and injected in a rat infarction model. A significant improvement in cardiac function in comparison to a PBS control was observed in terms of increase in ejection fraction and ventricular wall thickness, and a reduction in infarct size. Van Marion et al. (95) published promising results from the use of constrained and stress-free collagen/Matrigel systems to increase efficiency of cardiac stem cell therapy. Results showed that encapsulation of stem cells in these 3D gels stabilized cell viability and proliferation and moreover induced mechano-sensitivity. Recently, injectable conducting hydrogel systems have been reported (93). In 2017, a novel conductive hydrogel based on collagen, alginate, and a soluble non-toxic polypyrrole (PPy) was described (96) as a promising candidate for cardiac muscle regeneration. Due to incorporation of PPy, high conductivity, good cardiomyocyte viability, and syringe-ability were achieved. Although these developments show the potential of bio-hybrid gels in improving the efficacy of cardiac stem cell therapy, future clinical validation is needed to convert promising formulations into medically proven products.

### *In vitro* Engineering of Cell-Populated 3D Constructs

In the second strategy, the characteristic 3D tissue engineering approach, collagen is used to provide the *in vitro* 3D cellular support (in gel or solid form) for both *in vivo* and *in vitro* applications. *In vivo* usage includes implantation of the designed cell populated biomimetic construct (cardiac patch) on the infarcted myocardium to deliver healthy, functional cardiomyocytes to the damaged area of the heart, thereby enhancing the intrinsic regenerative ability of the host. The cardiac patch is expected to be remodeled and incorporated into the native cardiac tissue. *In vitro* applications include biomedical studies, generation of healthy cells for cell-based therapy, functional cell differentiation from stem cells, drug screening, and research into the development of new treatments.

### Collagen Hydrogels as 3D Cardiac Patches

The EHT, as described in the section above, was the first attempt at creating 3D cardiac patches with collagen. It exemplifies the efficacy of 3D gels in supporting cardiac cell activity within artificial *in vitro* models. The EHT work and other similar investigations (97, 98) demonstrated the possibility of creating 3D constructs, based on collagen gels and cells that develop, after culturing *in vitro*, structural, functional, and physiological characteristics similar to cardiac tissue. Another significant finding in these studies is that vascularization takes place in collagen gels when implanted *in vivo*. Unfortunately, mismatch of the mechanical and spatial characteristics of these gel-like systems with those of native myocardium currently precludes their clinical use. However, significant research effort has been focused toward their biomechanical properties and other key parameters. For example, an increase in the mechanical properties of collagen gels can be achieved by fibroblast-mediated compaction (99, 100). This phenomenon, first reported in the late 1970's (101) and whose mechanism is still not completely understood (99), has attracted extensive attention in the field of regenerative medicine and especially wound healing. Gel contraction increases collagen density and, consequently, mechanical strength. However, the extent of this contraction can be limited, and other more controlled methods need to be considered to reinforce gel mechanics to achieve desirable and predictable values for TE applications. For example, significant improvements in physical, mechanical, and biological properties, that are not readily achievable with individual collagen hydrogels, have been reported for hybrid silk fibroin-collagen gels (102). These include tuneable gelation time, stiffness levels covering important range of physiological values, excellent elastic behavior, and high resistance to cell mediated contraction. The incorporation of electroconductive components into collagen gel-like patches has also been considered as a means to enhance maturation and physiological properties of the engineered cardiac tissue by improving electrical coupling within, and between, the engineered graft and host tissue. For example, in 2018, Roshanbinfar et al. (93) reported a biohybrid hydrogel composed of collagen, alginate, and the electroconductive poly (3,4-ethylenedioxythiophene):polystyrene sulfonate (PEDOT:PSS) which, after having been seeded with neonatal rat cardiomyocytes, exhibited extracellular matrix–mimetic fibrous structures, enhanced electrical coupling and cardiomyocyte maturation. The presence of PEDOT:PSS in the hydrogel improved electrical conductivity and prevented arrhythmia of tissue constructs containing neonatal rat cardiomyocytes. Results demonstrate the potential of these electroconductive biohybrid hydrogels to be used for pharmaceutical drug screening or as *in vitro* produced tissues for the treatment of heart disease.

Currently, cell-populated collagen gels have demonstrated their potential as artificial cardiac models in a variety of *in vitro* applications (103, 104).

The use of collagen gels has also been investigated in differentiation and reprogramming approaches for the generation of functional cardiomyocytes *in vitro* (105). Successful stem cell differentiation into cardiomyocytes have been reported on collagen I and collagen V substrates (106, 107). It was also shown that direct as well as indirect reprogramming of fibroblasts into cardiomyocytes may benefit from the use of collagen gels (108, 109). The introduction of collagen I, for example, into fibrin-based hydrogels increased the percentage of contractile colonies out of the total number of cell colonies in direct proportion to the collagen type I content (108).

However, the low stiffness of gel-like systems and poor ability to create a spatial bio-mimetic environment somewhat limit their *in vivo* application. These restrictions may be overcome by development of solid porous 3D matrices, in which controlled porous morphologies and better mechanical characteristics may be achieved. This approach is described below.

### Prefabricated 3D Collagen Matrices

**Figure 4:**
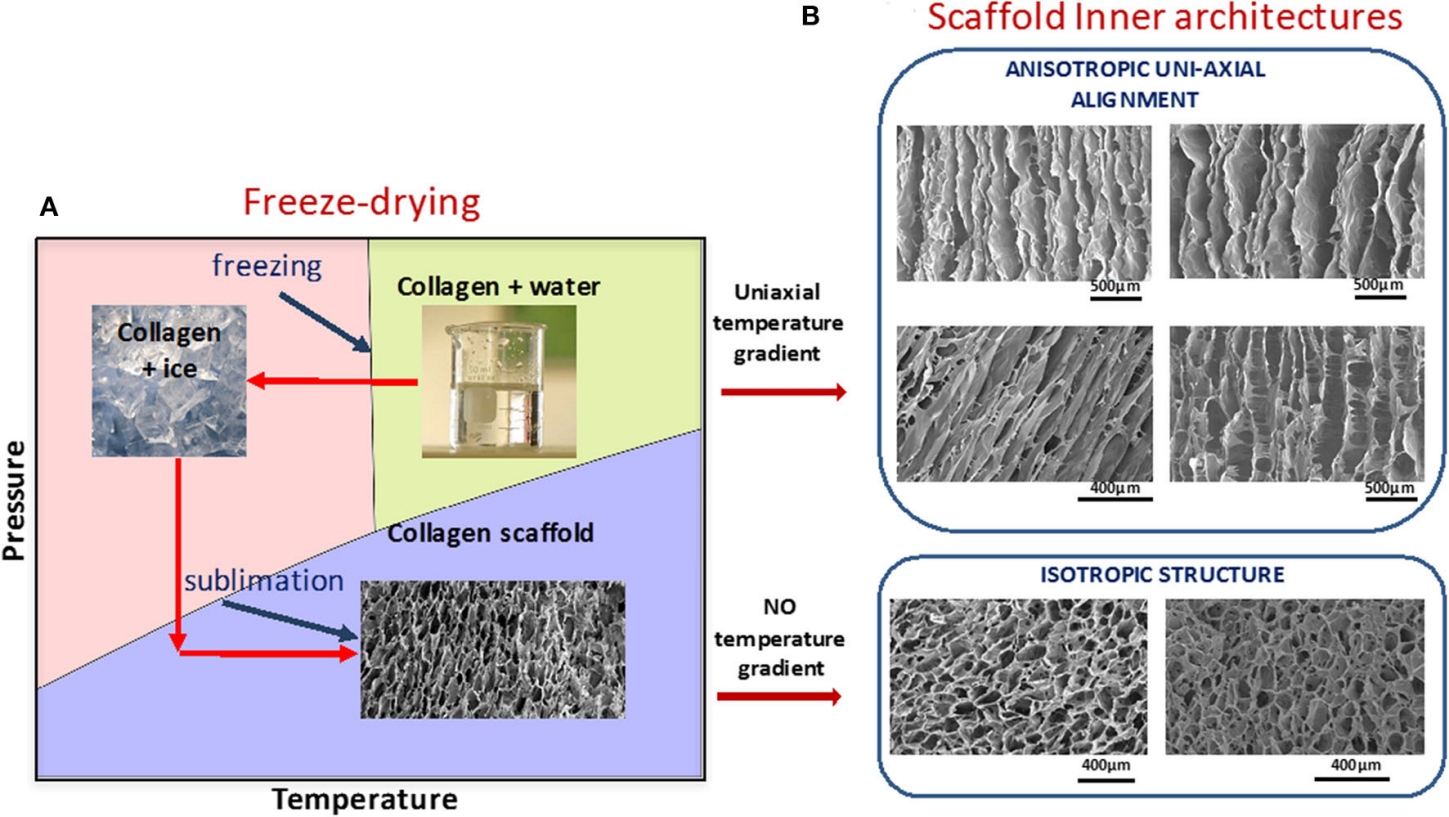
**(A)** Schematic representation of freeze-drying process. Ice structure leads to pore shape, size, and orientation. **(B)** Examples of different morphologies of collagen scaffolds. Anisotropy in the microstructures were achieved by imposing temperature gradients during the phase of crystallization of water in collagen suspensions, using molding technology. Images from Cambridge Center for Medical Materials, University of Cambridge, UK are part of Figure 7 from Davidenko et al. (113). License for re-using these images had been obtained from Copyright holder (Elsevier).

By selecting appropriate processing methods and conditions, collagenous scaffolds can be obtained with desirable structural morphology (pore size, interconnectivity, shape, and orientation), tailorable degradation kinetics, and tuneable mechanical characteristics (6, 71, 72, 110, 111). Special care should be taken during collagen processing to avoid denaturation. Among suitable technologies for engineering cell supports from naturally-derived collagen, a controlled freeze drying method represents one of the most successful procedures (6, 67, 71, 112). In this technique, the polymer suspension is cooled below its freezing temperature, forming an interconnected network of ice crystals, subsequent sublimation of which leads to the creation of a porous scaffold with an inner morphology that mirrors the structure of ice (Figure 4A). Pore size in an isotropic scaffold is controlled by the time at equilibrium (68) during freezing which is influenced by freezing parameters. These include freezing temperature, cooling rate, and temperature gradient and these strongly influence ice crystal morphology and, consequently, spatial architecture of the resultant scaffold. Anisotropy can be introduced by controlling temperature gradients in the freezing slurry (114). By using this approach, collagen matrices with controlled, and complex pore orientation that closely mimic many normal multi-oriented tissue arrangements have been produced (69, 113, 115, 116). Figure 4B shows some examples of different scaffold morphologies achieved (68, 113) by inducing uniaxial temperature gradients in collagen slurries during the scaffold fabrication stage.

**Figure 5:**
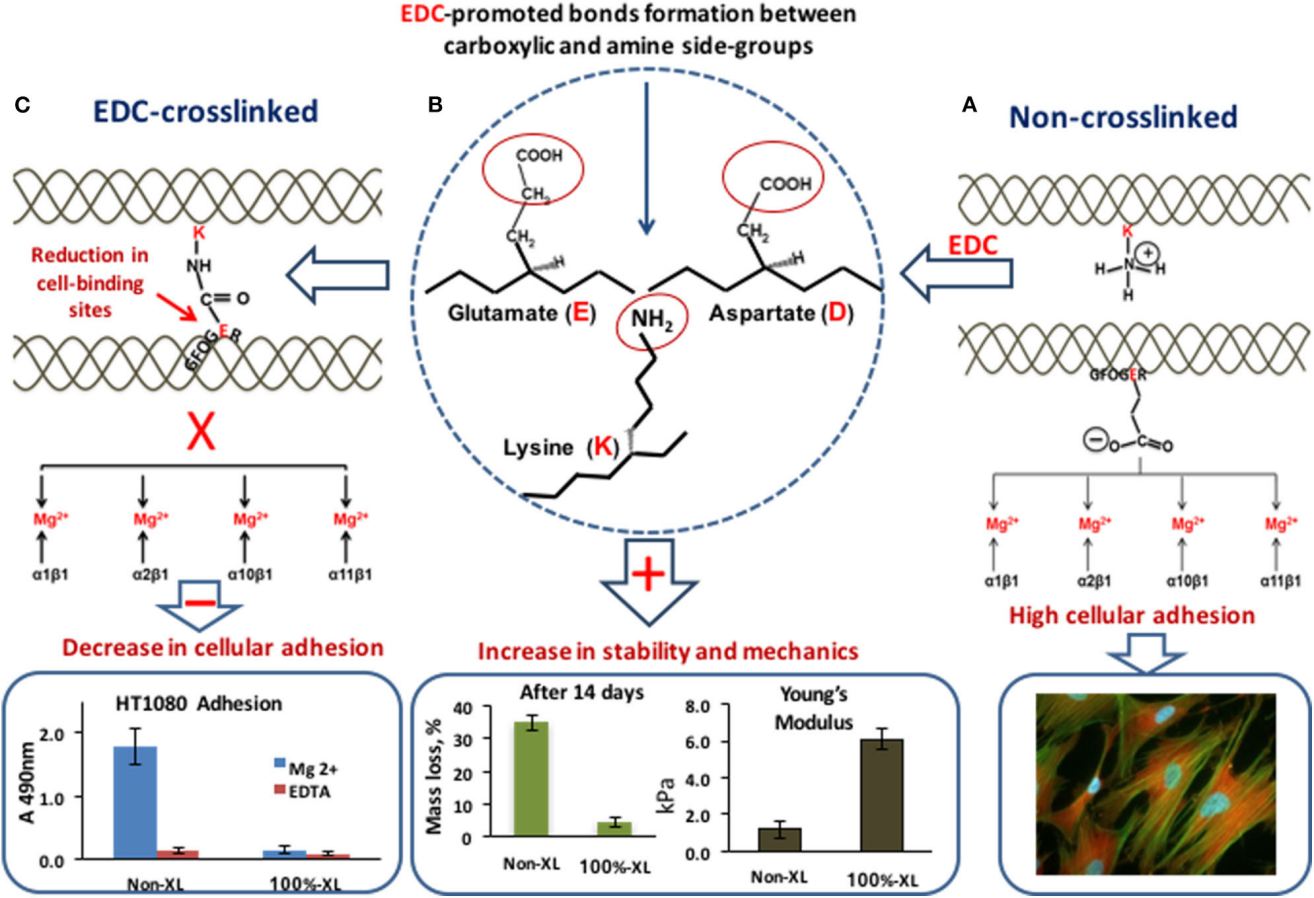
EDC-crosslinking. **(A)** In non-XL collagen two adjacent collagen helices: with a lysine (K) amine-containing sidechain and with the integrin-binding motif GFOGER with its crucial glutamate acidic (E) side chain. The carboxylate anion is free to coordinate a Mg^2+^ ion bound to the integrin α-subunit I domain, so that α1β1, α2β1, α10β1, or α11β1 can secure cell binding to the matrix. High cell adhesion. **(B)** EDC promotes the cross-linking of the glutamate (E) and aspartate (D) carboxylate group with the adjacent lysine (K) amine group. **(C)** Amide bond formation between adjacent collagen helices. The glutamate sidechain can no longer interact with integrins. EDC-crosslinking leads to the increase in scaffold stability to degradation and mechanical properties but affects the number of cell-binding sites with a negative effect on cell attachment. Data for graphs in the figure were replotted from Davidenko et al. (72) and Davidenko et al. (78).

To achieve a desirable biological performance from engineered collagen matrices, other key parameters, such as availability of cell binding ligands, swelling profiles, degradation rates, and mechanics should be finely tuned. Different physical (117–120) and/or chemical (71, 121–126) procedures can be used to provide strength and durability to collagenous matrices. Among them, carbodiimide (EDC)-based crosslinking (65, 71, 72, 127) constitutes one of the most successful, and as such, one of the most used tools for restoration of collagen cross-linking density, lost during its extraction and purification. However, EDC-promoted bonding has a significant drawback in that it uses carboxylate anions (for example the glutamate residue, E, of GFOGER), essential for integrin-mediated cell attachment (Figure 5), which may impinge on scaffold bioactivity (72, 78, 113, 128). To preserve or restore collagen native chemistry, different research strategies have been developed including the optimization of reactant crosslinking concentration [to reduce the loss of cell-reactive carboxylate anions (72)] and the attachment to crosslinked collagen of novel cell-adhesive peptides, designed to control, guide, and re-establish collagen biological activity after crosslinking (129, 130).

3D prefabricated matrices require appropriate seeding densities and homogeneously distributed cells to ensure electrical connection across the scaffold. Current approaches include the use of Matrigel as a vehicle for rapid cell delivery into collagen sponges. This, in conjunction with the immediate establishment of alternating-flow perfusion enabled rapid and spatially uniform cell seeding at densities close to physiological densities, while maintaining cell viability (131). Other strategies include the application of moderate centrifugal force during cell seeding resulting in uniform cell distribution (132).

Technological achievements in processing methods and acquired expertize in modulating essential properties of collagen-based matrices, have led to the development of promising formulations successfully employed in a variety of TE approaches. For example, at the beginning of the 2000s, Kofidis et al. (133) reported the use of collagen sponges for seeding of neonatal rat cardiomyocytes. The resultant artificial tissue, generated after *in vitro* cell culturing, possessed structural, mechanical, physiological, and biological characteristics similar to the native myocardium. Later, the same group investigated collagen mesh scaffolds (134) populated with undifferentiated embryonic stem cells for *in vivo* implantation into the infarct area of rat hearts. It was revealed that embryonic stem cells in these scaffolds formed stable intra-myocardial grafts that were incorporated into the surrounding area without distorting myocardial geometry, thus preventing ventricular wall thinning. Collagen type I sponges were also seeded with neonatal rat heart ventricular cell fractions. These cells developed contractile properties and were able to survive in these matrices, *in vitro*, for up to 135 days (135). In the subsequent investigation of the same group (136), collagen-I scaffolds were directly sutured to healthy or injured left ventricles of mice without previous *in vitro* cell culture. Encouraging results in terms of vascularization, scaffold degradation, and foreign body reaction have been reported. In a study by Xiang et al. (137), scaffolds formed from type I collagen and GAGs were seeded with adult bone marrow–derived mesenchymal stem cells and implanted into infarcted regions of rat hearts. Degradation rate and structural stabilities of these matrices were manipulated by crosslinking showing that EDC-treated scaffolds retained their sponge-like architecture through the entire implantation period, providing structural support to the failed regions of the heart. In a more recent study, collagen matrix was embedded with bone marrow cells and then transplanted into the patient with left ventricular post-ischemic myocardial scars. At 10 months after implantation clear improvement in the patient's condition was observed: left ventricular end-diastolic volume beneficially decreased and left ventricular filling deceleration time significantly improved (138). These effects were attributed to both the enhancement of cellular retention at the site of tissue injury and to the improvement of biological performance of cells in 3D substrates. It has been shown that the appropriate 3D environment of collagen scaffolds enhances the lineage differentiation capacity of stem cells (139–145) with a subsequent increase in cell therapeutic potency (141, 144, 146). The importance of an appropriate 3D microenvironment was also confirmed when 3D collagen type I scaffolds were used as artificial models of cardiac tissue for *in vitro* generation of functional cardiomyocytes from mesenchymal stromal cells (67). It was observed that collagen templates enhanced cellular differentiation into cardiomyocytes, increasing expression level of cardiomyocyte-specific proteins. Interestingly, the positive effect of collagen sponges was mostly attributed to their tri-dimensionality and biomimetic mechanical properties rather than to biochemical cues for inducing MSC differentiation. Additional stimuli for cardiomyocyte generation can be provided by electrical and mechanical stimulation in bioreactors and microfluidic devices (2, 104).

The results described above show the potential of collagen in creating artificial constructs with ECM-mimetic characteristics in terms of chemical composition, spatial architecture, and physical and mechanical properties, suitable for hosting cells, supporting attachment, proliferation, and cell-guided tissue formation. This results in successful *in vitro* models of cardiac tissue for different TE approaches. However, there are still many challenges to overcome before *in vitro* generated cardiac implants, be they built from collagen or other natural or synthetic material, are converted into clinically effective products. One of these challenges is associated with a difficulty of designing scaffolds that have nonlinear elasticity similar to the heart muscle and thus develop synchronous beating with the recipient heart (2–4). Other challenges are related to vascularization which is crucial for adequate mass transport, cell survival, electromechanical integration, and functional efficiency of the transplanted cardiac patch (2, 147). Advances in these key areas will allow translation of successful *in vitro* formulations into effective therapeutic tools.

## Alginate

**Figure 6:**
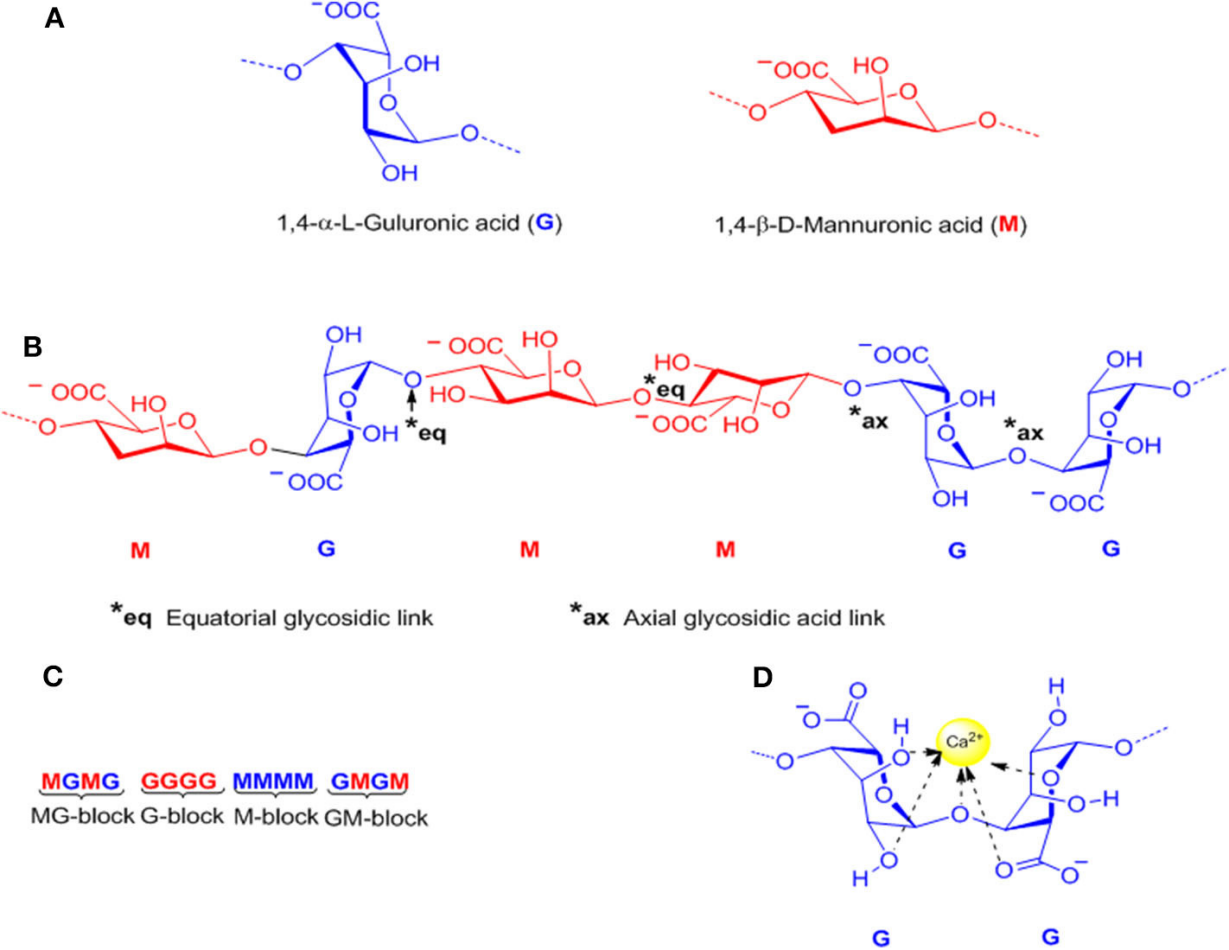
Representative alginate structure: **(A)** Monomers, **(B)** Chain conformation, **(C)** Block distribution (M-block, G-block, and MG or GM block), and **(D)** Schematic model of hydrogel formation “egg-box model”.

Alginates are a group of natural polysaccharides that are considered to be biocompatible, biodegradable, non-toxic, and non-immunogenic (148, 149). Alginates were discovered in 1881 by a British pharmacist E.C.C Stanford, while exploring novel and useful products from kelps (150). In 1896, algin was properly isolated by Krefting Kelco Co. (151) in California, but it was not until the end of the 1950s that industrial production of alginates was expanded to Europe and Japan (152). The composition and sequence of alginate copolymers consist of 1,4-linked*-*β*-*D-mannuronic acid (M block) and 1,4-α-L-guluronic acid (G block) units (Figure 6A) interspersed in regular (poly-G, poly-M) or irregular blockwise pattern of varying proportions of GG, MG, and MM blocks (153) (Figures 6B,C). The M block segments provide the linear and flexible conformation of the main backbone chain due to a linkage in diequatorial position, β(1–4) mannuronic acid for the MM blocks, whereas the G blocks serve to introduce folded and rigid structural conformation by a steric hindrance around the carboxyl groups, and the existence of a linkage in the diaxial position for the GG blocks, α(1–4) guluronic acid, responsible for a remarkable stiffness of the polymer chains. Figure 6 shows the chemical structure of alginates (154).

The chemical and physical properties of alginates are affected by structural parameters such as the monomer composition, sequential structure, and molecular weight of the polymeric chain. Also, depending on the source and species that produce the copolymer, alginates, can be obtained with a wide range of molecular weights (between 32 and 400 kDa) (155–157).

### Alginate Production Methods

Alginate production can be carried out via bacterial biosynthesis since alginates are exopolysaccharides produced by several bacterial strains including *Azotobacter* and *Pseudomonas aeruginosa* (158). The biosynthesis involves the oxidation of a carbon source to acetyl-CoA, which via gluconeogenesis is converted into fructose-6-phosphate (F6P) during the Krebs cycle (159, 160). However, commercial production of alginates is based on an extraction process from different marine macroalgae, brown algae, also called seaweeds, *Macrocystis pyrifera, Laminaria hyperborean*, and *Ascophyllum nodosum* (161, 162). Particularly, the seaweeds commonly known as kelps (order Laminariales) are most widely used as common raw material for alginate production worldwide (163–165).

### Hydrogel Formation

Alginates have a number of free –COO^−^ and COOH acid groups which are responsible for their water solubility and suitability for chemical functionalization (166). Alginates can be easily converted to hydrogels by using cross-linking agents such as calcium ions (Ca^2+^) (Figure 6D). The coordination of the divalent ions is through the junctions of the G blocks of one polymer with other G blocks of adjacent polymer chains, known as the “Egg-box-model” (167) (Figure 6D). The gelation of alginate is a chemo-reversible process, a property that is quite useful to form cell-immobilization matrices (168–170). One critical drawback of this cross-linking method is the rate of degradation and the stability of the alginate hydrogel in physiological conditions. In this sense, the covalent cross-linking offers a permanent method of gelation, and also, allows the possibility to control degradation rates and mechanical stiffness using an appropriate cross-linking agent and by controlling the degree of cross-linking (171–173). Since mammals lack the alginase enzyme, alginate is a non-degradable material, however, the partial oxidation of alginate chains promotes degradation under physiological conditions.

### Alginate-Based Biomaterials for Cardiac Tissue Engineering

The scope of the applications of alginates in the field of biomedicine is broad and includes cell transplantation, delivery systems of drugs, and proteins; wound healing, among other applications (155, 174). The non-thrombogenic nature of the alginates is one of the most attractive properties and makes it an ideal material for cardiac applications (132, 175–177). Such applications involve the use of alginate hydrogels and porous 3D scaffolds, and focus on four major areas including: (1) extracellular matrix (ECM) substitute in heart tissues to promote tissue regeneration due the structural similarity between alginate and natural heart ECM, (2) delivery system for cardiac stem cells or adult cardiomyocytes to the injury sites, (3) platform for sustained delivery of growth factors to mimic the natural physiology, and (4) gels to control drug release (178).

### Alginate Hydrogels as Extracellular Matrices

The application of alginates as extracellular matrices is generally carried out through direct local injection into the infarcted myocardium or via intracoronary injection. Direct injection of an alginate gel into the infarcted myocardium of rats demonstrated a persistent improvement of the left ventricular (LV) fractional shortening and prevention of continued enlargement of the LV dimensions (179). However, alginate hydrogels have a poor bioresorbability and low cell adhesiveness, which may lead to adverse tissue interaction and poor regenerative properties (180). The alginate modification with cell adhesion ligands such as arginine-glycine-asparagine (RGD) can promote the cell-matrix interaction. Yu et al. carried out a comparative study using the neat alginate hydrogel and alginate modified with Arg-Gly-Asp (RGD) in cardiac repair. The alginate hydrogel reshaped a dilated aneurismal LV and improved LV functions, whereas the RGD modified alginate enhanced the angiogenic response (181). Subsequent studies conducted by the group of Randal tested the efficiency of the alginate hydrogel implants (Algisyl-LVR^TM^) in dogs with heart failure (HR) induced by repetitive coronary microembolization (182). During an open chest surgery, the final injection (a mixture of sodium-alginate aqueous solution with calcium cross-linked alginate hydrogel) was applied directly into the LV wall. The treatment was well-tolerated. Four-month post-treatment, histological analysis showed that the material was encapsulated by a thin layer of connective tissue with no evidence of an inflammation reaction. Compared to the control (saline-treated animals), the alginate implantation significantly increased the ejection fraction (EF) from 26% at baseline to 31%, wall thickness, improved the LV sphericity, and reduced the LV diastolic and end-systolic volume as well as end-diastolic pressure. These promising results led to the initiation of clinical trials for intramyocardial delivery of alginate implants, under the name Algisyl®, in patients with an enlarged acute LV myocardial infarct (MI). The implant is administered directly into the LV wall using 19 injections (177). In addition, alginate was shown to reduce the wall stress of the dilated heart and prevent further dilatation and negative LV remodeling, even in human hearts (183). Recent studies have shown a persistent effect of LV augmentation of Algisyl in humans at 12-month post-treatment, a clinically relevant improvement in exercise capacity and symptoms was observed for patients with advanced HF (184). On the other hand, an injectable alginate was developed by Landa et al. (185) which could be delivered by intracoronary injection as an aqueous solution. This solution was a mixture of calcium cross-linked alginate with calcium gluconate solution. Biotin-labeled alginate was used for temporary tracking of the injectable material and injected into the infarcted area 7 days after anterior myocardial infarction. Due to high calcium concentration at the acute infarct site and the water diffusion from injectable solution to the surrounding tissue, the gelation process occurs *in situ*. The alginate hydrogel was replaced by host tissue within 6 weeks after the administration. Echocardiography studies showed that injection of this biomaterial reduced LV dysfunction, diastolic, and systolic dilatation. Other studies have proven the beneficial therapeutic effects of this novel *in situ* forming alginate hydrogel in acute myocardial infarction (MI) model in pigs (186) and in acute and chronic models of myocardial infarction in rats (185).

### Alginate as Immobilization Matrix for Cardiac Cells

As previously mentioned, the innate physical properties of alginate hydrogel facilitate cell retention and they are most commonly used for intramyocardial delivery of mesenchymal stem cells (MSC). Several studies have shown that alginate can provide the required temporal support for cell growth and function as an artificial biomimetic ECM, until the cells are able to support themselves (187, 188). However, in contrast with other studies, Karpov et al. showed that practically all embedded cells in pure alginate die prior to capsule degradation. Additionally, a non-significant reduction in the scar size between non-encapsulated and encapsulated cells was observed compared to those in the control MI (189).

As we mentioned above, the incorporation of ECM-derived peptides into the alginate hydrogel enables cell adhesion and other functions, further maturing the seeded cells. The RGD peptide is a commonly used alginate modifier because it is derived from the laminin and fibronectin signal domain. Often the peptide-cell interaction could be specific to certain types of cells; however, RGD-peptide modified alginate is versatile since the peptide mediates the cell adhesion and signaling between ECM proteins and integrin receptors on the cell surface (190).

**Figure 7:**
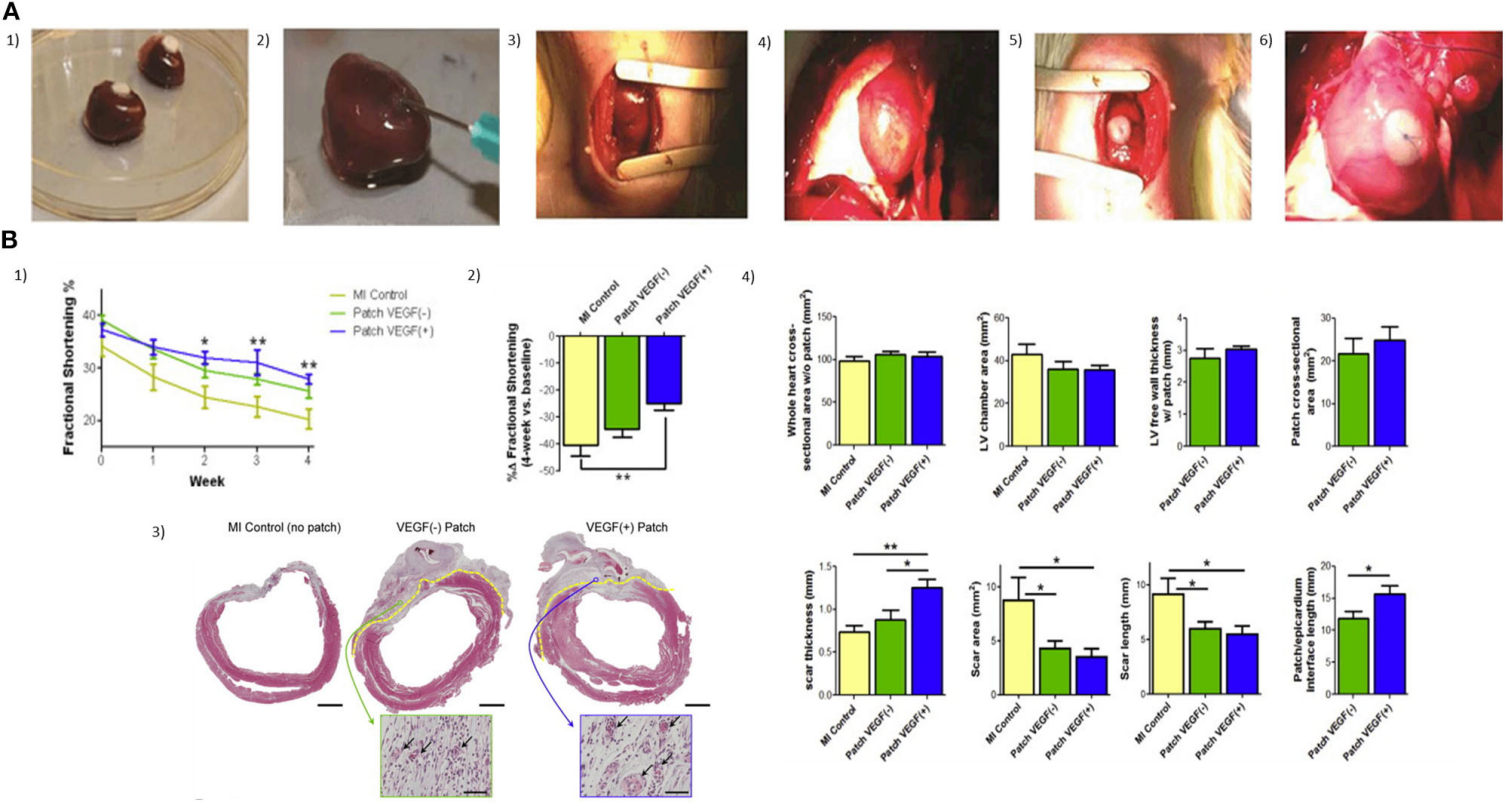
**(A)** Two injectable gels (chitosan and alginate) and two epicardial patches (collagen β-glycerophosphate and alginate) were compared in terms of acute retention of stem cells in the infarcted heart (1, 2). Injection technique and volume, patch size, and attachment were optimized with rat hearts *ex-vivo*; (3) Mini-thoracotomy and guide suture placement; (4) Myocardial blanching was observed after ligation of the LAD; (5) Patches were placed at the infarct border zone cell-seeded side down with a single suture; (6) Patches remained in place for 24 h, when a bilateral thoracotomy was performed and aorta was cannulated for perfusion (191). **(B)** Epicardial microsphere patches improve cardiac functioning and VEGF^(+)^ patches improve cardiac morphometry post-MI. (1) Myocardial infarction (MI) was induced in mice by left anterior descending artery ligation. Patches were transplanted onto the LV surface of the heart 4 days after MI, and fractional shortening (% FS) was measured for 4 weeks; (2) To compensate for variability at baseline (1-week post-MI, pre-implantation, *t* = 0) FS was also expressed as a percentage change over the 4-week time course (%Δ FS); (3) Tissue morphometry was assessed using Masson's trichrome stain. Patch/epicardial interference were identified under high magnification and are indicated with a broken yellow line (scale bar = 2 mm). Insets show vascular structure (arrows) in the patch areas (scale bar = 50 μm); (4) Left ventricular and patch morphometry were quantified using whole-slide scanned trichrome stained cross-sections (192).

Roche et al. tested RGD-modified alginate hydrogels and chitosan-β-glycerophosphate as delivery systems for improving MSC retention in a rat MI model and epicardial patch (191). In comparison to the saline control, treated hearts exhibited a significant increase in cell retention after 24 h (9% vs. 50–62% cell retention; Figure 7A). Levit et al. (193) encapsulated human mesenchymal stem cells (hMSCs) in alginate hydrogel and then attached it to the heart with a poly(ethylene glycol) (PEG) hydrogel patch, in a rat MI model. Hydrogels were detectable up to 2 weeks after implantation but fully degraded by 28 days. *In vivo* bioluminescence imaging showed higher retention of cells in animals treated with encapsulated hMSCs compared to delivery by direct injection. hMSCs were only visualized in non-cardiac tissue in the direct injection group, suggesting that minimal washout or migration from the gel and capsules occurred. A total increased microvascular density and a significantly decreased scar size were observed after 28 days.

Injection of RGD-modified alginate microspheres with and without MSCs in a 1-week rodent model of MI, led to improvement in the preservation of wall thickness, fractional shortening, and LV internal diameter—wall thickness with MSCs alone decreased from 2.5 ± 0.1 to 1.9 ± 0.3 mm over 10 weeks post-injection, but with microspheres alone it was maintained from 2.8 ± 0.3 to 2.8 ± 0.5 mm, and with the MSCs in microspheres it went from 2.6 ± 0.2 to 2.5 ± 0.4 mm. *In vivo* experiments with immunodeficient nude rats demonstrated that at 2 weeks post-injection, the microspheres still indicated good retention of cells (0.532%). Echocardiography performed at 10 weeks post-injection demonstrated an improvement in LV function of microsphere injected groups (194). The conjugation of the RGD peptide into macroporous alginate scaffolds increased functional cardiac muscle tissue formation and improved the preservation of the regenerated tissue properties in long-term *in vitro* cultures (195). An alginate scaffold modified with the synthetic cyclic Arg-Gly-Asp-D-Phe-Lys (RGDfK) peptide was recently reported by Sondermeijer et al. (196). The porous scaffold was generated using a novel silicone sheet sandwich technique in combination with freeze-gelation. The cyclic RGDfK peptide is protease-resistant, highly stable in aqueous solution, and has a high affinity for cellular integrins. These novel scaffolds sufficiently adhered to the myocardial surface without sutures, and significantly higher cell retention than unmodified scaffold was observed. A lower initial seeding density on RGDfk-modified scaffolds showed significantly more vascularization at the infarct border zone than scaffolds without cells 1 week after transplantation, increasing the LVFS (4.7%) compared to saline controls. Surprisingly, an opposite effect was observed at a higher dose of hMPSCs. The overcrowding stress may explain this effect. Sondermeijer et al. estimated the production cost of 1 RGDfk-modified alginate scaffold to be around US$ 1500 (size 100 mm × 0.75 mm using 2% RGDfk-modified alginate), excluding cells and culture materials. Although the production cost was relatively cheaper compared to other biomaterials, more studies should be carried out over extended periods of time in order to know its potential and feasibility in clinical trials.

In addition, macroporous scaffolds made from pristine alginate modified with RGD and heparin-binding peptide (HBP), made by the freeze-dried process, displayed a greater stiffness and stability in culture, compared with the conventional alginate hydrogel. hESC-CMs and human dermal fibroblasts (HFs) were seeded in macroporous scaffolds in serum free, chemically defined medium. The addition of fibroblasts to the 3D culture allowed the formation of functional cardiac tissues and the presence of peptides attached to the alginate scaffold further improves its functionality. By day 35, the polarization of the connexin-43 to the CM membrane edge indicated improved maturation of the cardiac tissue (197).

Exosomes are tiny microvesicles released by cells in response to different physiological states. Their ability to carry cell type-specific mRNA and miRNA, both implicated in the regulation of multiple biological processes, result in them playing a principal role in cell-cell communication (198, 199). Exosomes, from various types of stem cells, can mimic the effect of their original parent cell, also they have high stability in biological fluids. Hence, exosomes have become an attractive strategy for clinical applications in critical illness.

Exosomes secreted by resident adult cardiac progenitor cells (CPCs)(CD9+, CD63+, CD1+, heat shock protein 70+, Alix+, and tumor susceptibility gene 101+) are effective in cardioprotection and repair of infarcted hearts (200), Cellular uptake of exosomes is quick, resulting in rapid dissemination of the vesicular contents to the target cells. Therefore, an important area for consideration is the long-lasting beneficial effects after delivery and strategies for enhancing their therapeutic activity.

Exosomes loaded in alginate-based hydrogels might be considered in this area for preserving the exosomes in the wound site and acting as an extracellular matrix. Monteforte et al. (201) reported the use of alginate hydrogels loaded with glioma-derived exosomes to enhance revascularization in peripheral ischemia. Alginate beads with exosomes induced angiogenesis *in vivo* showing their potential therapeutic effect for isquimia. Also, alginate-based hydrogel loaded with exosomes was recently proposed as a novel therapeutic approach to skin tissue engineering. Its impact was compared with alginate-based hydrogel and conventional sterile gauze on the full-thickness excisional wound in a rat model. The application of hydrogel loaded with exosomes greatly enhanced wound closure, reepithelization, collagen deposition, and angiogenesis at the wound site (202). Undoubtedly, these results open up a host of opportunities for exploring alginate-based hydrogel loaded with exosomes in the cardiovascular field.

### Hybrid Hydrogel

In order to improve the interaction and response of cardiac cells to various stimuli patterns, 3D nanocomposites have been studied as scaffolds for cardiac tissue repair. 3D macroporous nanocomposites of gold nanowires with alginate improved the electrical communication between adjacent cardiac cells, enhancing the cell organization, synchronous contraction under electrical stimulation, and higher expression level of sarcomeric α-actinin and Cx-43 on day 8(203). Another interesting approach for cell delivery involved alginate-based cardiac patches with magnetically responsive nanoparticles (204), which were exposed to an external magnetic stimulation at a physiologically relevant frequency (5 Hz) to determine whether the addition of nanoparticles would promote the formation of myocardial tissue. Neonatal rat cardiac cells seeded within these novel scaffolds were subjected to magnetic stimulation which resulted in a more mature myocardial tissue characterized by anisotropically organized striated cardiac fibers that preserved the desirable features for a longer time than non-stimulated constructs at 15 days of cultivation. A high activation rate of AKT phosphorylation in cardiac cell constructs was detected after applying a short-term 20 min external magnetic field, indicating the efficacy of magnetic stimulation to actuate at a distance. These results showed a synergistic effect of magnetic field stimulation together with nanoparticulate features as providing the regenerating environment for cardiac cells driving their organization into functionally mature tissue. In the same way, Hao et al. (205) reported an injectable scaffold based on fullerenol nanoparticles/alginate hydrogel as a cell delivery vehicle with antioxidant activity. Brown adipose-derived stem cells (BADSCs) were seeded in fullerenol/alginate hydrogel and their biological behavior in the presence of H_2_O_2_ was studied. Results suggested that the nanocomposite hydrogels have no cytotoxicity effects on BADSCs and also, they can suppress the oxidative stress damage of the cells, improving their survival capacity under reactive oxygen species (ROS) microenvironment via activating the p38 and the extracellular-signal-regulated kinase (ERK) pathway while inhibiting the c-Jun N-terminal kinase (JNK) pathway. Also, *in vivo* studies showed that the injectable fullerenol/alginate hydrogel can effectively decrease the ROS level in the MI zone and improves the retention and survival of implanted BADSCs and induces angiogenesis. The retention and survival in the fullerenol/alginate group are significantly higher than in the pure alginate hydrogel group.

Exploring new approaches for cell maturation, a conductive hybrid hydrogel composed of collagen, alginate, and poly(3,4-ethylenedioxythiophene): polystyrene sulphonate (PEDOT:PSS) was developed by Roshanbinfar et al. to analyse the contractile behavior of engineered cardiac tissue. A nonconductive hybrid hydrogel (CA-gel) (collagen and alginate) exhibited arrhythmic contraction at a frequency of 8–21 beats min^−1^ between day 5 and 11 and stopped after 13 days. Surprisingly, the conductive hydrogel, composed by collagen, alginate, and 0.26% w/w PEDOT:PPS (eCA-gel, ionic conductivity of 27 ± 8 × 10^−4^ S cm^−1^), exhibited spontaneous rhythmic beating with frequencies increasing from around 22 at day 5 to 220 beats min^−1^ at day 11. High beating frequencies of eCA-gels were detected until day 13, and spontaneous contraction was still detected at day 40. Non-significant difference in response was observed between eCA-gels and CA-gels to external electrical stimuli at 1 and 5 Hz. Also, orientation maps and graphs showed that cardiomyocytes are oriented unidirectionally in eCA-gels (93).

### Controlled Growth Factor Release From Alginate-Based Matrices

Growth factors, cytokines, and stem cell-mobilizing factors are bioactive molecules of high interest in the field of therapeutic myocardial regeneration due to their potential in cell proliferation, vascularization, apoptosis inhibition, progenitor cell differentiation, and progenitor cell migration (206, 207). Hao et al. (208) used an alginate hydrogel consisting of both high and low molecular weight hydrogel, also known as binary molecular weight alginate, for studying the sequential delivery of vascular endothelial growth factor (VEGF) and platelet-derived growth factor (PDGF)-BB into myocardial infarction. VEGF is an important initiator of angiogenesis associated with improvements in cardiac revascularization of the infarcted myocardium (209) and induces protection of cardiomyocytes against ischemic death. Zentilin et al. explored the effects of VEFG-A and VEGF-B_167_ in cardiomyocytes exposed to hypoxia. The percentage of apoptotic cells dropped from 17.2% of controls to 7.6 and 8% in the VEGF-A and VEGF-B treated cultures, respectively, when cardiomyocytes were exposed for 90 min to the cardiotoxic drug epirubicin (210). The same effect was obtained from CellBeads containing human mesenchymal stem cells (MSCs) during the treatment of critical limb ischemia (CLI). Through secretion of VEGF-A from CellBeads, an increase in the muscular blood flow and oxygenation was observed around the site of administration (211). However, delivery of this growth factor alone may lead to immature and leaky vasculature with poor function (212), hypotension, proteinuria, and cardiac toxicities, among other serious adverse effects (213–215). Given this consideration, alginate-based matrices become an appropriate delivery system for this purpose. The cumulative release of VEGF-A_165_ and PDGF-BB from alginate hydrogels *in vitro* following incubation in PBS at 37°C showed that 80% of the growth factors were released at 30 days. Seven days after the MI was induced in rats, the alginate hydrogels loaded with the factors were injected intra-myocardially, along the border zone of the infarct. Four weeks after injection, the slow sequential growth factor administration led to a higher density of alpha-actin-positive vessels (mature) than with a single factor. The sequential protein delivery enhanced the systolic velocity-time integral and displayed a superior effect than the single factors. Also, alginate microspheres have been applied successfully for growth factor release in cardiac application due to their prolonged release and tuneable degradation properties. Rodness et al. (192) combined the approaches of microsphere properties and cardiac patches to produce a compacted calcium-alginate microsphere patch, supported by a chitosan sheet to deliver VEGF to the heart after MI in rats. The microsphere patch-treated hearts showed better cardiac function than the unloaded chitosan patch. However, histological studies showed an essential difference between VEGF (+) and VEGF (–) patches. VEGF (+) patched hearts had thicker scars characterized by higher capillary density in the border zone than those treated with VEGF (–) patches (Figure 7B).

### Alginate Based-Drug Delivery System

Alginates are widely used in the pharmaceutical industry as gels, matrices, membranes, nanospheres, microspheres, and coating material (216). Their chemical and degradation properties make alginates an ideal candidate for local drug deliveries including drugs used to treat cardiovascular diseases. Lovich et al. (217) developed epicardial drug-releasing hydrogels for applying dobutamine, an ionotropic agent for use in congestive heart failure, to the left ventricle of rats. Epicardial dobutamine increased indices of contractility with less rise in heart rate and lower reduction in systemic vascular resistance than IV infusion. Alginate polymers are also useful for administration of poorly water-soluble drugs. A promising system to enhance drug dissolution rate and maintain drug supersaturation levels in the gastrointestinal fluid was developed by Franca et al. (218). Solid dispersions of chlorthalidone were prepared by spray drying using sodium alginate as carrier and sodium lauryl sulfate or polyvinyl caprolactam-polyvinyl acetate-polyethylene glycol graft copolymer (Soloplus), as surfactants. At sink condition, formulations showed a faster dissolution rate than the crystalline drug. On another hand, the formulation and the coating composition of biopolymeric pellets containing ranolazine, an anti-angina drug, were studied by Segale et al. (219). Coated-alginate pellets were prepared by ionotropic gelation using different concentrations of hydroxypropyl cellulose (HPC) and alginate. The rate and the entity of swelling process were affected by the polymeric composition, with the increase in the HPC concentration, the structure of the pellets became more compact, slowed down the penetration of fluids, and determined a slower release of the drug.

Finally, alginates have also been applied successfully in potential treatment for other cardiovascular diseases such as 3D printed aortic valves (220, 221), in blood vessel engineering (222, 223) and as a direct antihypertensive (224–226).

Alginate has proved its potential and applicability in the pharmaceutical and biomedical field due to its versatile favorable characteristics. The most critical features of alginate for this application include non-toxicity, biocompatibility, and mild gelation process. However, despite the extensive research of alginate properties and progress made in cardiac applications, most of their potential remains unexplored. Future investigations on alginates may focus on the design of new classes of alginate with precisely designed and chemical properties which might respond to different stimuli and ensuring synergistic effects of alginates on cardiac tissue engineering.

## Silk as a Scaffold Biomaterial for Cardiac Tissue Repair

**Figure 8:**
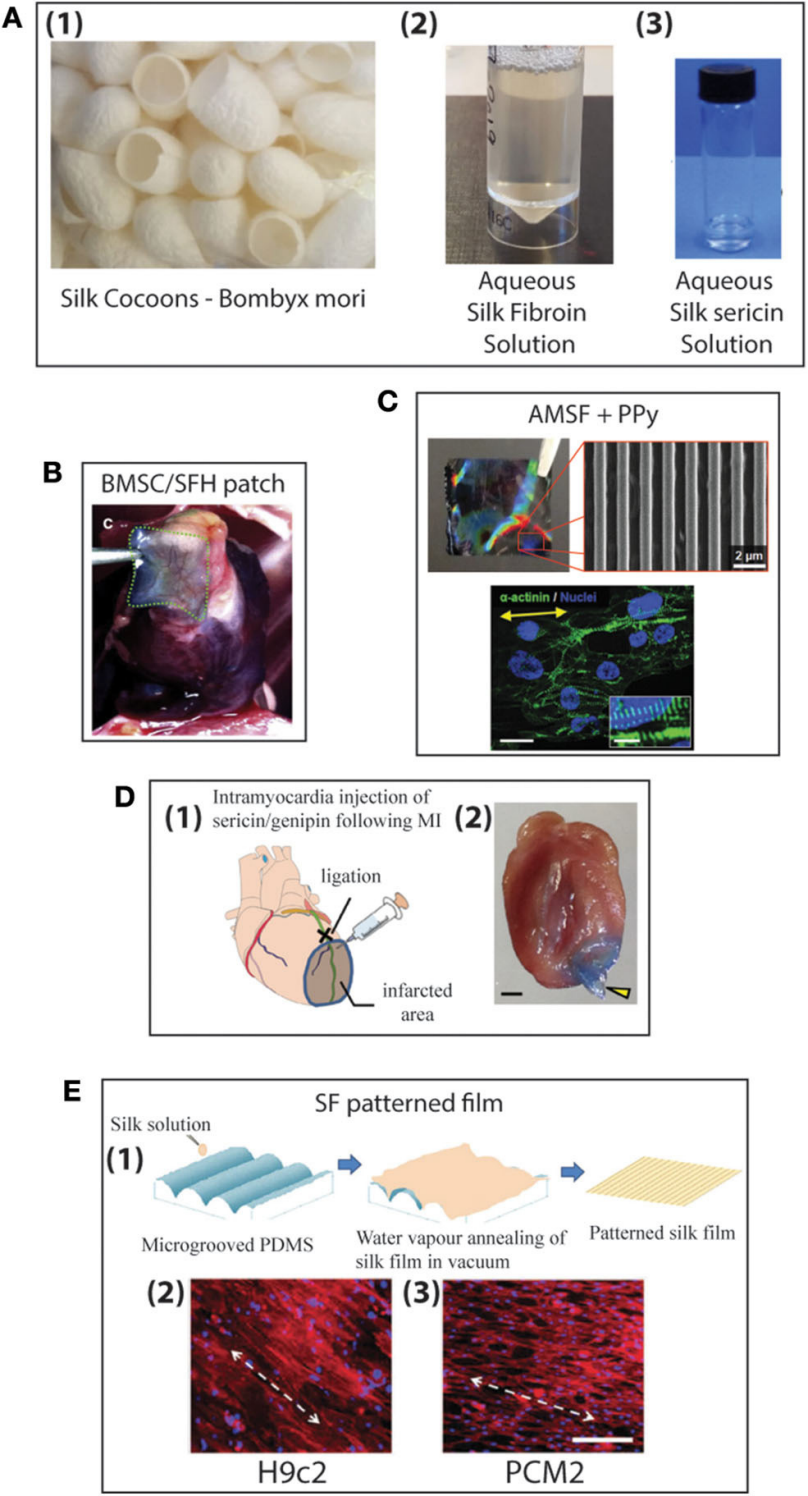
**(A)** Production of aqueous silk solutions from silk cocoons (1), Fibroin solution (2), Sericin solution (3) **(B)** Histological image of MI zones of heart for Bone marrow mesenchymal stem cells/silk fibroin/hyaluronic acid (BMSC/SFH) patch shown after 8 weeks of infarction (233). **(C)** Nanopatterned silk substrate of nanopatterned acid-modified silk fibroin (AMSF) with deposited poly(pyrrole) (PPy) (1 cm^2^). SEM image of AMSF + PPy nanopatterned substrate. Cardiomyocytes fluorescently stained for α-actinin (green) and nuclei (blue). Cells on nanopatterned substrates exhibit elongated and aligned morphologies. Yellow arrows indicate the direction of the nanopattern. Scale bar: 25 mm; inset 10 mm (234). **(D)** Genipin crosslinked sericin hydrogel (1) schematic showing the anatomical site (black cross) of the occlusion of left anterior descending coronary artery (LAD) (green line), the corresponding infarcted myocardial region (shaded area), and the injection site of the sericin/genipin hydrogel delivered via a syringe. (2) Macroscopic view of a wild-type heart with a layer of myocardium at the LAD-supplied area cut to open showing an *in situ* forming of genipin-crosslinked sericin hydrogel (yellow arrowhead). Scale bar, 1 mm (235). **(E)** Schematic representation of the fabrication of patterned silk films using microgrooved PDMS molds (1). Biocompatibility of silk films with cardiomyocytes: fluorescent microscopy images of confluent monolayers displaying unidirectional alignment of H9c2 (2) and Primary ventricular cardiomyocytes (PCMs) (3) on patterned silk films. Actin cytoskeleton (red: Rhodamine–phalloidin), nucleus [Hoechst 33342 (blue)]. White arrows indicate the direction of the alignment (scale bar−200 mm) (236).

For centuries humans have harvested silk from silkworms to produce clothing and as sutures. Most commonly, silk fibers are recovered from the cocoons of the silkworm *Bombyx mori*, however spiders also produce silk which is researched for its superior mechanical properties (227, 228). At present a variety of silk-based biomaterials have been approved by the FDA and therefore make silk a desirable material to be used in biomedical applications. For many biomedical applications silk cocoons are initially degummed under boiling conditions in CaCO_3_ solution to remove the sticky sericin protein which accounts for around 30% of the silk weight. The remaining mass (~70%) is accounted for by the silk fibroin (SF) protein and it is this that is mostly used for tissue engineering applications. For further processing the silk fibers are dissolved in either Ajisawa's Reagent (Ethanol: CaCl_2_: Water) (229) or Lithium bromide (230, 231) to produce a clear regenerated silk fibroin (RSF) solution also known as Silk I. Silk is known to exist as three polymorphs, these are Silk I: a glandular state before crystallization, Silk II: a spun silk state consisting of its β-sheet secondary structures and Silk III: an air/water assembled interfacial form with a helical structure (227, 232) (Figure 8A). Silk I is the commonly used polymorph to create a variety of biomaterials as this is a water soluble form of silk and can be easily converted into Silk II by exposure to different conditions such as heat, shear force, and a variety of solvents or salt solutions. Some of the first reports dating back to 2012 for the use of silk fibroin (SF) in the treatment of myocardial infarction (MI), were reported by Chi et al. (233), where they create a SF/hyaluronic acid (HA) patch containing bone marrow mesenchymal stem cells (BMSCs) in a rat MI model. The BMSC/SF/HA patches were tested for a duration of 8 weeks and found to be well-adhered, intact, and showing little to no immunological responses (Figure 8B). They significantly enhanced the survival of BMSCs and prevented the apoptosis of cardiomyocytes as well as stimulated the secretion of important growth factors for cardiac repair. The literature reveals that in the last few years, there has been an increasing interest in the development of silk-based scaffold materials (234–243). For example, Tsui et al. (234) produced electroconductive acid-modified silk fibroin–poly(pyrrole) (AMSF + PPy) scaffolds patterned with nanoscale ridges to enhance structural and functional properties of cultured human pluripotent stem cell (hPSC)-derived cardiomyocytes (234) (Figure 8C). The authors reported an enhanced organization of cardiomyocytes, *in vitro*, exhibiting improved sarcomere and gap junction development as well as increased expression of genes that are part of the control for cardiac tissue excitation and contraction functions. To show the diverse tuneability of silk-based biomaterials in another study Song et al. (235) demonstrated the promotional effect a silk/sericin hydrogel has on the cardiac functional recovery after MI. The authors show how the *in situ* crosslinking silk gel lead to an increased micro-vessel density and myocardial recovery as well as a reduced inflammatory response and attenuated apoptosis in the infarcted region, leading to an improved functional recovery. As the gel crosslinks *in situ* the whole process is less invasive than other patch methods, however, it is worth noting that the degradation dynamics reported indicated a total hydrogel degradation after ~21 days which is much shorter compared to other more rigid silk scaffold materials (Figure 8D).

When looking at natural biomaterials it is important to look at different aspects such as the species producing the silk but also the food they consume and how these can affect their produced biomaterials and reproducibility (244). It is in this context that in contrast to others Mehrotra et al. (236) report a comparative study of SF patterned monolayers produced by *Bombyx mori* and *Antheraea*. The silk films were produced by water vapor annealing under vacuum, cast on a microgrooved Polydimethylsiloxane (PDMS) mold (Figure 8E). The authors found that the non-mulberry silk scaffolds from *A. assama* exhibited better mechanical strength and elasticity as well as a lower immunogenicity and better compatibility to cardiomyocytes compared to the *B. mori* scaffolds. In another interesting study Petzold et al. (243) demonstrated the use of recombinant spider silk protein eADF4(κ16) in *Araneus diadematus* to overcome previously reported reproducibility issues. They reported an engineered modified sequence of ADF4, where the glutamic acid residue of the repetitive unit was replaced with lysine in the core domain of the SF. In this study, films were produced by dip coating glass substrates into an eADF4(κ16) solution and then letting the solvent dry off naturally. Here, no patterning of the films was considered, and *in vitro* studies of cardiomyocytes grown on eADF4(κ16) films in comparison with fibronectin films was investigated. The cardiomyocytes responded well to pro-proliferative factors as well as exhibiting good cell-to-cell communication and electric coupling similar to fibronectin films. The authors indicate the potential ability to print the eADF4(κ16) silk solution without the need of additional crosslinking agents for future cardiac applications, along with the potential for further genetic modification to further optimize the functionality and processability (233).

Thus current literature indicates that silk-based composite materials can be used to form excellent tuneable scaffold materials for cardiac repair with low immunological responses, good cell adhesion, and proliferation, as well as superior mechanical properties (234, 242, 243). Silk as a biomaterial gives the opportunity to create a variety of materials including fibers (237), foams (241), hydrogels (231), nanoparticles (245), films (243), and 3D printed structures (246, 247). It also has tuneable degradation rates as well as the potential for gene and drug delivery in the created constructs.

## PHAs: Natural Polymers of Bacterial Origin

**Figure 9:**
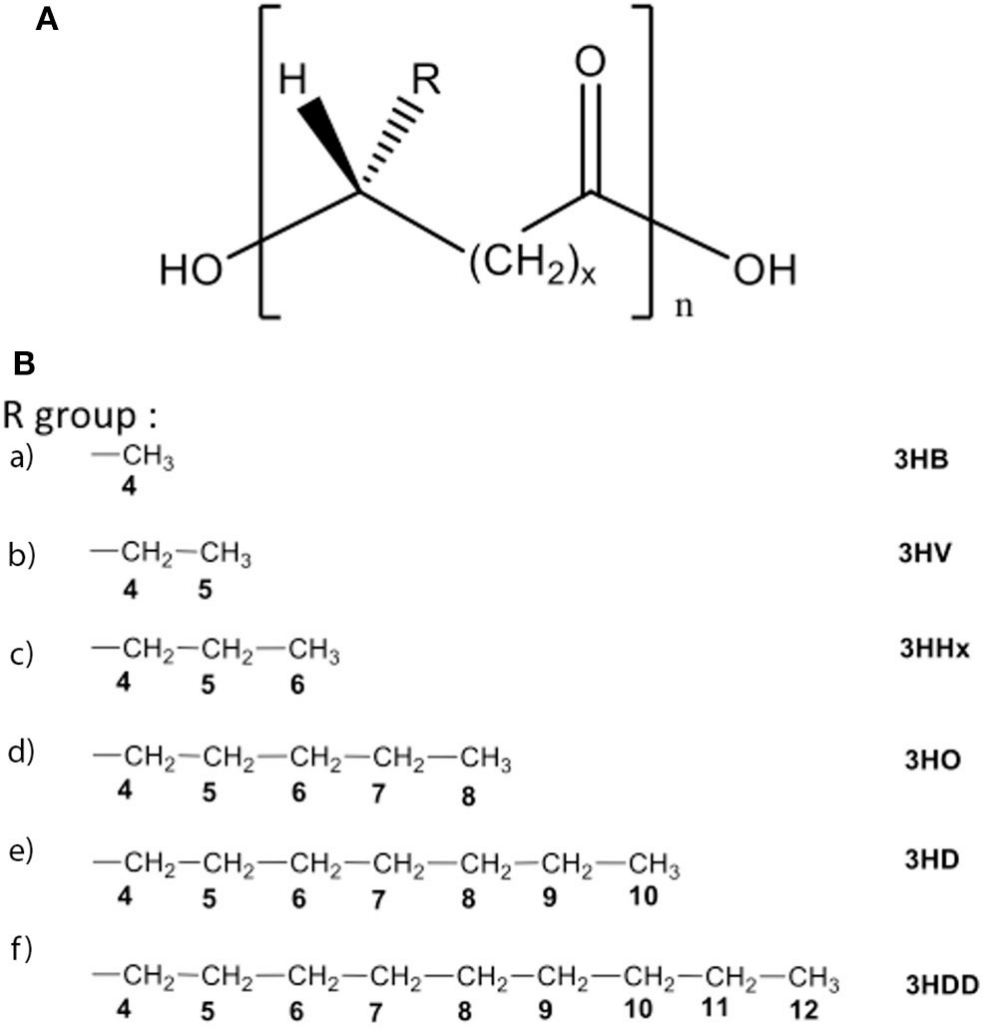
**(A)** The general structure of PHAs. **(B)** The R groups of various PHAs that have been utilized in cardiac tissue engineering. The short chain length PHAs (SCL-PHAs); monomers include 3HB: 3-hydroxybutyrate and 3HV: 3-hydroxyvalerate whilst for medium chain length PHAs (MCL-PHAs) that have been investigated monomers include 3HHx: 3-hydroxyhexanoate, 3HO: 3-hydroxyoctanoate, 3HD: 3-hydroxydecanoate and 3HDD: 3-hydroxydodecanoate.

While clear improvements in the mechanical and other functional properties have been made for the natural materials described so far, they do not approach the range and flexibility of synthetic polymers. A bridge between these two worlds is given by polymers produced by bacterial fermentation. Derived from the monomers of 3-,4-,5-,6-hydroxyalkanoic acids, Polyhydroxyalkanoates (PHAs) are a family of bioresorbable aliphatic polyesters normally produced using fermentation of bacteria under nutrient limiting conditions. Characterized by their monomer composition, PHAs are classified in to two main types, short-chain length PHAs (SCL-PHAs) and medium-chain length PHAs (MCL-PHAs), each with unique properties. SCL-PHAs contain 3–5 carbon atoms within their monomer unit whereas MCL-PHAs are produced from monomers containing 6–16 carbon atoms in their monomer unit (Figure 9). The bacterium and the carbon source used for the fermentation are crucial in determining which of these two subsets of PHAs are produced.

Differences in the length of the monomer units results in variation in the mechanical properties of these two polymer subsets. Due to their larger monomer units, the side chains of MCL-PHAs do not readily pack closely together, therefore these polymers are highly flexible elastomers that exhibit low crystallinity and in turn, a low glass transition temperature (T_g_). These properties make MCL-PHAs such as Poly(3-hydroxyoctanoate) (248, 249), Poly(3-hydroxyoctanoate-*co*-3-hydroxy-decanoate) (250, 251), ideal for soft tissue engineering (STE) such as cardiac applications (252). Conversely, SCL-PHAs are generally semi-crystalline, brittle polymers with high melting temperatures (T_m_). Poly(3-hydroxybutyrate, P(3HB) (253), the best-studied SCL-PHA, holds great potential for hard tissue engineering applications. However, whilst also a SCL-PHA, Poly(4-hydroxybutyrate), P(4HB), is highly elastomeric and exhibits an elongation at break (E_b_) of 1000% and has received FDA approval for its use as a suture material (254).

The large variability of monomer units (C_3_-C_16_), in addition to the variability in the position of the hydroxyl group results in numerous configurations of PHAs, each with bespoke mechanical characteristics. This is in contrast to conventional synthetic copolymers such as Poly(lactic-co-glycolic acid) (PLGA) where only the mole% of the two respective monomers can be adjusted to modify the polymer characteristics. To this end, the highly crystalline nature of P(3HB) makes it difficult to biodegrade and thus its biomedical applications have been limited (255). To address this, numerous copolymers of P(3HB) have been generated including Poly(3-hydroxybutyrate-*co*-4-hydroxybutyrate), P(3HB-*co*-4HB), Poly(3-hydroxybutyrate-*co*-3-hydroxyhexanoate), P(3HB-*co*-3HHx), and Poly(3-hydroxybutyrate-*co*-3-hydroxyvalerate), P(3HB-*co*-HV), which are less crystalline than the homopolymer and also have an increased E_b_, thus enhancing the potential medical applications of P(3HB). Additionally, P(3HB) has been shown to become more crystalline and brittle when aged. Conventionally, plasticizers are added to polymers to modulate their mechanical properties, however, these are often at the expense of biocompatibility. Recently, oligomers of MCL-PHAs derived via hydrolysis were shown to reduce the crystallinity of P(3HB), thus reducing stiffness, whilst not compromising on biocompatibility (256).

Although highly tuneable, the side chains of PHAs do not conventionally contain polar groups such as hydroxyl or carboxylic acid groups. As a result, PHAs are relatively hydrophobic in nature, therefore they degrade via surface erosion rather than bulk degradation, as observed for conventional, synthetic polymers including PLGA. As such, PHA derived tissue engineering scaffolds have the potential to maintain their structural integrity over a longer period, thus aiding the endogenous timeline of tissue repair. Upon their degradation, PHAs release weak hydroxy acids with high p*K*a values (4.70 and 4.72 for 3- and 4-hydroxybutyric acid, respectively), in contrast to the relatively stronger acids, lactic acid (p*K*a 3.86), and glycolic-acid (p*K*a 3.87), the degradation products of PLGA (257).

In addition to being weaker acids, thereby less likely to instigate an inflammatory response, the degradation products of PHAs are often naturally occurring metabolites found *in-vivo*. For example, 3-hydroxybutyric acid, the breakdown monomer of P(3HB) is a ketone body found within blood plasma and urine (258) whilst P(4HB) degrades into 4-hydroxybutyric acid [γ-Hydroxybutyric acid (GHB)] which is naturally found in numerous organs including the heart and skeletal muscle (259) and can be clinically administered for treatment of neurological disorders (260).

The biocompatibility of PHAs has been demonstrated in various *in-vivo* studies using medical grade PHAs. Microspheres and tubes derived from the co-polymer Poly(3-hydroxyoctanoate-*co*-3-hydroxyhexanoate), P(3HO-*co*-3HHx) were subcutaneously implanted into a mouse model and although a thin layer of fibroblasts was observed at 2 weeks, this did not increase over time (40 weeks) and no macrophages were identified during this period (261). Subsequent studies of P(4HB) derived films implanted subcutaneously in rats also revealed a minimal immune response (262).

**Figure 10:**
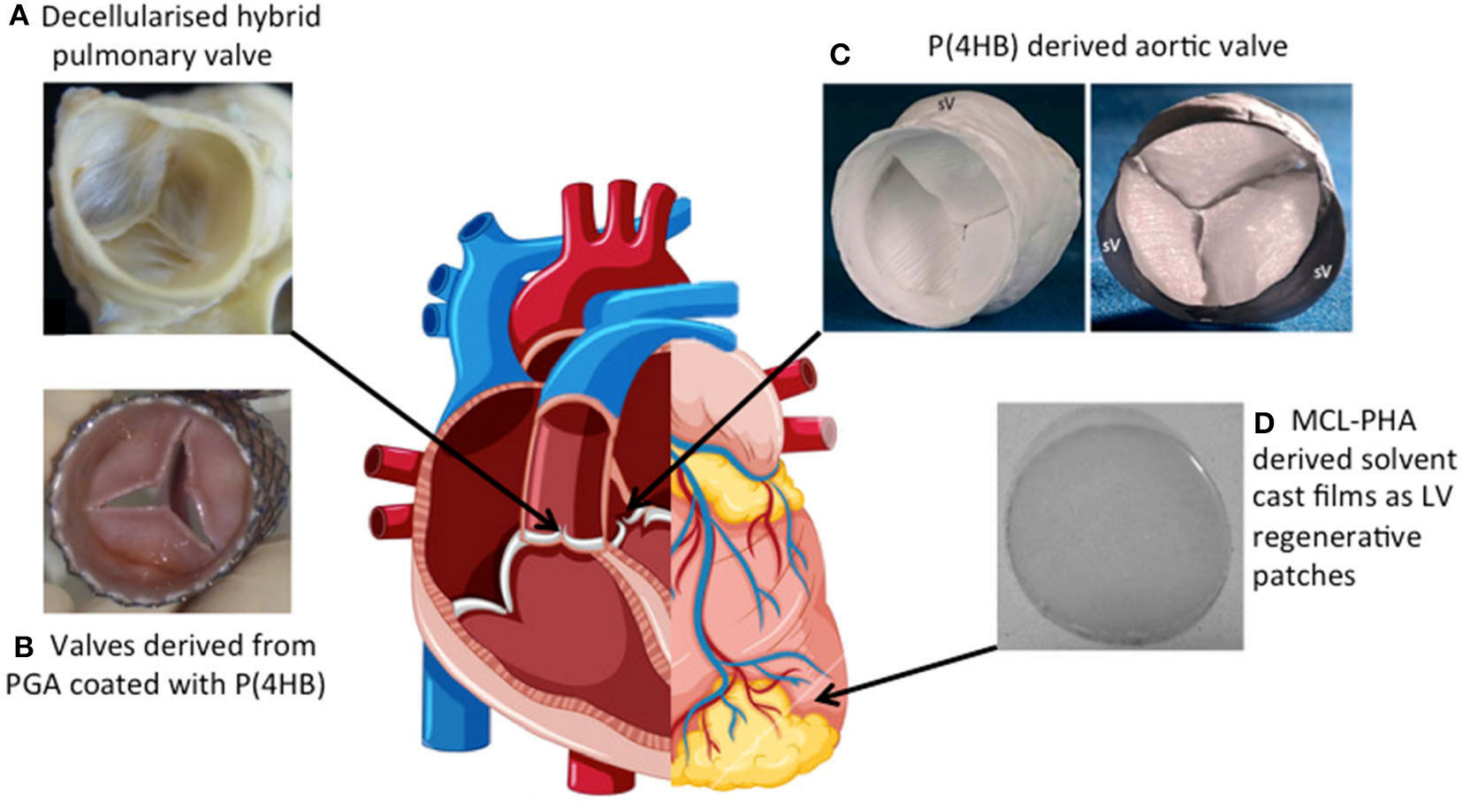
Macroscopic images of **(A)** Decellularized porcine valves impregnated with P(4HB) and implanted into the pulmonary position of sheep for 12 weeks display viability and retain the overall structure of the valve (263). **(B)** A tissue engineered heart valve derived from PGA, coated with P(4HB) and subsequently seeded with either adult stem cells or vascular cells. It has been placed into a self-expanding stent and is to be delivered to the pulmonary position of sheep for 8 weeks (264). **(C)** Aortic grafts derived solely from P(4HB) following molding using a CT generated structure of a human aortic valve. SV highlights the Sinus of Valsalva (265). **(D)** Solvent cast film derived from MCL-PHAs that has been utilized for LV cardiac regeneration (266, 267).

Due to their excellent biocompatibility, in addition to their diverse mechanical properties, PHAs have been assessed for various aspects of cardiac tissue engineering. Valve replacements (Figures 10A–C) and regenerative cardiac patches (Figure 10D) have been investigated, either through fabrication of complete scaffolds or as coatings to facilitate the functionalization and mechanical properties of decellularized organ homo-/xeno-grafts or other polymer derived grafts.

### Left Ventricular Regenerative Patches

Numerous studies have investigated PHAs, namely P(3HB), for their suitability as anti-adhesive pericardial patches (268, 269), to be used following cardiac surgery to prevent adhesions, or for artery augmentation (270). More recently, PHAs are beginning to be evaluated for their suitability as substrates for regeneration of the myocardium following MI.

During diastole, the Young's modulus of the human myocardium is 0.02–0.5 MPa (271). The mechanical properties of MCL-PHAs are not vastly dissimilar and are therefore well-suited biomaterials for LV regeneration. Furthermore, their high processability allows for the fabrication of complex 3D structures, containing defined anisotropic structural cues, potentially capable of maturing hPSC-CMs toward a more adult phenotype.

Despite being a brittle SCL-PHA, electrospun P(3HB) fibers were compared to fibers produced from other well-studied natural and synthetic biomaterials. Although all of the investigated biomaterials were shown to be biocompatible with a range of cell types including the cardiac line HL-1, P(3HB), (alongside PCL) displayed superior adhesion and growth of cells when compared to other natural biomaterials like collagen and silk fibers. Acellular fibrous scaffolds derived from these biomaterials were implanted into a rat model of MI. Both P(3HB) and collagen scaffolds commenced degradation within 8 weeks of implantation without evidence of encapsulation of the scaffold. Rather, these scaffolds were able to instigate the M2 macrophage phenotype which is often associated with enhanced repair post-MI. This was in contrast to silk and PCL fibers that were encapsulated following an M1 macrophage response. Of the scaffolds investigated, P(3HB) was also shown to facilitate improved angiogenesis as determined by a greater number of capillaries and arterioles in both the healthy and infarcted myocardium. Although these effects manifested in reduced scar formation and a prevention in ventricular dilation, none of these acellular scaffolds were able to improve systolic function as assessed via echocardiography 2 weeks post-implantation (272).

As a result, other PHA scaffolds have been generated and assessed with the addition of cells. Indeed, the elastomeric MCL-homopolymer P(3HO) has been assessed for its potential as a LV post-MI regenerative patch. Analysis of its mechanical properties revealed a Young's modulus of 3.7 MPa reduced to 1.5 ± 0.4 MPa at 37°C followed by a further reduction upon the introduction of porosity to 0.41 ± 0.03 MPa. Although marginally greater than that of the adult myocardium, this can be beneficial in preventing post-MI cardiac hypertrophy and myocardial remodeling. A high degree of elasticity was reported at body temperature (699.3 ± 113%), essential for the beating of the heart. Furthermore, CM adhesion, cell viability, and proliferation of C2C12 myoblasts on P(3HO) scaffolds was shown to be comparable to that of collagen, despite the hydrophobic nature of P(3HO) and the lack of prior preconditioning with ECM proteins. Given its processability, P(3HO) was electrospun to generate fibers whilst the incorporation of the RGD-motif, known to enhance cell attachment as well as the incorporation of vascular endothelial growth factor (VEGF), further improved cell proliferation of the C2C12 myoblasts, thus highlighting the potential of MCL-PHAs for cardiac regeneration (266).

Furthermore, a 95:5 wt% blend of MCL-PHA/PCL has been seeded with murine atrial derived cardiac progenitor cells (CPCs), a heterogeneous population of cells containing, endothelial, fibroblasts, and cardiac “stem cells.” To address the poor cellular retention following administration via intra-myocardial injection, porous films were generated from the blend as a means of delivering CPCs to the myocardium, allowing for their release in a controlled fashion. Introduction of PCL resulted in a reduction in hydrophobicity, thus enhancing cell adhesion. Cells were tracked *in-vivo* via (^19^F) MRS, showing a reduction in cell density on the scaffold across a 9-day period perhaps as a result of the cells detaching from the scaffold and entering into the myocardium (267).

In addition to aiding the delivery of CMs to the myocardium, PHAs have been investigated for their role in the differentiation and maturation of CMs. Shijun et al. (273) compared the cardiomyocyte differentiation efficiency of mouse iPSC (miPSC) cultured on 2D and 3D Poly(3-hydroxybutyrate-co-3-hydroxyhexanoate), P(3HB-co-3HHx) relative to TCP. Although both the 2D and 3D films of P(3HB-co-3HHx) were capable of superior miPSC adherence and proliferation relative to the TCP control, the 3D film was able to deliver enhanced cardiomyocyte differentiation efficiency (273).

Further investigation of stem cell derived CMs was conducted by Dubey et al. (274) through the assessment of hiPSC-CMs cultured on either P(3HO) or poly(3- hydroxynonanoate-co-3-hydroxyheptanoate), P(3HN-co-3HHP) films relative to TCP.

Cellular viability was determined to be upwards of 90% following 2 weeks of hiPSC-CM culture on films derived from these PHAs. Subsequent functional assessment of these cells was completed by way of beating measurements and calcium handling analysis. P(3HN-co-3HHP) was characterized as a highly elastomeric biopolymer relative to gelatin and as hiPSC-CMs displayed an increased beating rate relative to TCP controls. Furthermore, the time to peak calcium release as assessed by optical mapping was quicker for cells cultured on P(3HN-co-3HHP) relative to either P(3HO) or TCP control. Although no difference in sarcomere length was reported for hiPSC-CM cultured on films derived from either of these PHAs, cellular alignment was reported following the culture of hiPSC-CMs on electrospun fibers derived from either PHA (274).

These studies highlight a promising future for PHAs in the field of myocardial regeneration. Subsequent studies will aim to generate PHA-derived 3D tissue mimics of the LV complete with intrinsic structural cues and a range of cells capable of facilitating *in vitro* maturation of CMs followed by their *in vivo* retention. Although the use of PHAs for myocardial repair may be in its infancy, the diversity of mechanical properties observed in the PHA family is in stark contrast to other biomaterials and as such, has seen PHAs utilized for a range of cardiac tissue engineering applications with cardiac valve replacement perhaps being the best-studied.

### Cardiac Valve Replacement

In addition to the development of patches for the treatment of MI, CTE holds great potential in the treatment of valvular heart disease. Damage to the cardiac valves results in leaking or stenosis of the valve and as such, the current gold-standard treatment is to replace the defective valve with a mechanical substitute. Although highly durable, they are poorly biocompatible and present a high risk of stenosis and thrombus formation, hence the need for life-long anti-coagulation therapy.

Tissue engineering of heart valves (TEHV) is an alternative strategy being investigated, however, a number of challenges including the complex anatomical architecture of the tissue, the mechanical flexibility of the leaflets in response to physiological flow and pressure, and a surface that is free from stenosis, embolism, or generation of abnormal blood flow must first be addressed.

One way to recapture the complex architecture of the valve is to use a homo- or xeno-graft. To reduce the immunogenicity presented by these grafts, decellularization protocols can remove the pro-immunogenic elements of the tissue, leaving behind the complex architecture and the extracellular matrix (ECM). The ECM plays a key role in valve homeostasis by preventing stenosis and thrombosis whilst also facilitating flow. Upon decellularization of the valve, however, collagen fibers within the ECM are often damaged. Not only is their exposure highly thrombogenic due to their activation of platelets, it also reduces the mechanical strength of the graft. PHAs have been investigated to address these limitations through either their incorporation into decellularized grafts or the complete generation of valvular structures from PHAs.

Grabow et al. (275) investigated the generation of PHA-hybrid valves by impregnating aortic porcine valves with PHA or dip coating with either P(3HB) or P(3HB-*co*-4HB). Although the latter resulted in a solid polymer film, it was susceptible to delamination under physiological flexure whilst the impregnated xenograft was capable of generating a mean transvalvular pressure gradient comparable to that of the native valve. Decellularized valves were then impregnated with P(3HB) or P(3HB-*co*-4HB) resulting in reduced platelet activation *in vitro* relative to the uncoated xenograft, thus suggesting that collagen fibers were less exposed however the same was not observed for valves impregnated with P(4HB) alone.

*In vivo* assessment was conducted through implantation of these impregnated grafts into the rabbit abdominal aorta resulting in patent valves containing host vasculature and completing lining of the lumen with host endothelial cells. Although the P(3HB) impregnated valves were free of blood clots, there was some evidence of clotting in the P(4HB) hybrid grafts. Furthermore, a degree of calcification was observed in P(4HB) grafts. Despite this however, P(4HB) is less crystalline, more pliable than P(3HB) and also degrades quicker, therefore a 82:18% P(3HB-*co*-4HB) co-polymer was generated and assessed in a sheep aorta resulting in the migration of host smooth muscle cells into the leaflets and the generation of a confluent endothelial cell lining in addition to no evidence of stenosis (263).

Although polymer impregnation enhanced the mechanical properties of the decellularized xenografts (275) it did not recapture the microarchitecture of the ECM. Therefore Hong et al. (276) utilized the highly processable nature of PHAs and deposited P(4HB)-derived submicron fibers onto the surface of porcine aortic xenografts via solution electrospinning resulting in a fibrous network which also led to an enhancement of tensile strength and elastic modulus relative to the decellularized xenograft.

### PHA-derived Valvular Grafts

Although decellularized valves have the advantage of maintaining the 3D structure of the leaflets, the low availability of homografts coupled to the ethical considerations of using xenografts has resulted in researchers developing valves purely from biomaterials.

Owing to their thermoplastic properties, it was possible to mold P(4HB) and P(3HB-co-3HHx) into valvular structures. This was in contrast to the conventional biomaterial PGA which exhibited poor mechanical properties, including their stiffness and lack of pliability, resulting in an inability to fabricate functioning valves from this biomaterial, even when made into non-woven meshes (277). Similarly, it was also possible to mold valves using P(3HO) which were subsequently seeded with autologous ovine vascular- and endothelial-cells. Fabricated valves were implanted into the pulmonary artery of a lamb model where they remained viable for 17 weeks. Upon follow up, an increase in the inner diameter alongside the length of the valve was observed suggesting that the graft was growing with the animal. The study could not conclude whether this was true regeneration and growth of the valve or rather expansion of the construct. The valve did become more elastic over the 17 weeks resulting in a stress–strain curve resembling that of a native pulmonary artery valve. The M_w_ of polymer reduced by 30% during this period, potentially indicating that the construct was degrading and being replaced by tissue. Additionally, ESEM showed that following cell seeding the surfaces of the leaflets and conduit wall were smooth. This was in contrast to the non-seeded P(3HO) control which had not been endogenously populated, however despite this, there was no evidence of thrombus formation on the non-seeded control further illustrating the high biocompatibility of PHAs (278).

Given the highly processable nature of PHAs, it has been possible to mimic the complex architecture of the native aortic valve using computer topography (CT). An aortic homograft was scanned via CT resulting in the generation of a silicon mold onto which P(4HB) was molded, resulting in the production of a valvular construct with dimensions that deviated only 3–4% from the homograft (265). Such an approach relied solely on molding P(4HB), therefore did not require sutures, which are known to disrupt blood flow and cause thromboembolism, to attach the valve leaflets. A dripping technique was then used to seed the valves with myofibroblasts derived from the differentiation of cryopreserved human umbilical cord cells (CHUCCS). The cellularized valves were subsequently incubated in a dynamic bioreactor system that mimicked developmental conditions by gradually increasing pulsatile flow and pressure, resulting in an organized ECM and an enhanced tensile strength relative to static controls (279). CD133-positive cells were also isolated from umbilical cord blood and differentiated into myofibroblast and endothelial-like cells. The valves were populated with these cells again via a dripping technique followed by culture in a dynamic bioreactor. Myofibroblasts were seeded first forming a confluent layer of α-SMA expressing cells. The endothelial cells, seeded on top of the myofibroblasts, formed a monolayer that behaved like a functional endothelial network mimicking *in-vivo* characteristics as assessed by nitric oxide (NO) and intracellular calcium signaling, following acetylcholine and histamine stimulation, respectively (280).

The diversity of the cardiac applications in which PHAs have been utilized is testament to their excellent biocompatibility and processability. It has been possible to use a multitude of fabrication techniques to generate a number of bespoke structures using PHAs, both homopolymers, and co-polymers and have been selected over conventional biomaterials due to their superior mechanical properties. As PHAs continue to attract attention in the cardiac field, their potential as left ventricular regenerative patches will further be explored in conjunction with advanced fabrication techniques and stem cell-derived cardiomyocytes and endothelial cells.

## Active Factor Delivery Using Natural Biomaterials

In addition to the natural biomaterial cardiac repair techniques discussed in this review, extracellular vesicles and exosomes are also being researched for cardiac repair. Natural biomaterials can be utilized to deliver acellular biological components to a site of damage. For example, hydrogels can be used to encapsulate active factors and provide an injectable material for efficient delivery. A number of different hydrogels have been used in acellular cardiac repair research, including those based on alginate, chitosan, collagen, decellularized myocardium and pericardium, fibrin, fucoidan, hyaluronic acid, keratin, Matrigel, and PEG (281).

miRNAs are another active factor which has been researched in combination with hydrogels for cardiac repair. miRNAs are an alternative avenue of emerging therapeutic potential, as these can be used to stimulate repair mechanisms within tissues without the issues of cell transplantation. Specific miRNAs have been found to have a role in cardiac protection after acute myocardial infarction. miRNAs released by cardiac progenitor cells, including miR-17, miR-103, miR-210, and miR-292, have been shown to be pro-angiogenic and able to decrease the levels of profibrotic gene expression, aiding in the preservation of the myocardium's contractile function and therefore overall cardiac function (282). Others, for example, miR-30a, have been shown to increase post-myocardial infarction and have a role in the prevention of cell apoptosis (283).

The main obstacle with miRNA delivery is that they are degraded rapidly in the body due to the high quantity of RNases that are present in the body. Natural biomaterials can provide a solution for both the local delivery of miRNAs and enhancing their stability within the body for longer periods of time. In a study by Wang et al., an injectable hyaluronic acid-based hydrogel was used to encapsulate miR-302 for its local injection to the heart. They showed that this treatment enhanced the proliferation of cardiomyocytes in a mouse model, in a way that mimicked cardiomyocyte proliferation with miR-302 *in vitro*. Importantly, they found that in an MI mouse model, this injection improved the functioning of the heart (284). Previous research has shown the use of hydrogel biomaterials as a bioactive scaffold for the delivery and preservation of exosomes in wound sites. For example, a study by Shafei et al. (202) used an alginate-based hydrogel loaded with exosomes for a wound dressing application and found that it was biocompatible and biodegradable and increased wound healing in an animal model. Studies such as these show the huge potential for natural biomaterial hydrogels to be used as a biocompatible and bioresorbable delivery vehicle for exosomes that contain pro-angiogenic and anti-apoptotic factors that can aid in cardiac function restoration.

Another way of utilizing exosomes for cardiac repair is by using drugs to promote their release from cells that are either present in the injured myocardium or being used for cell therapy. A recent study by Casieri et al. (285) investigated the regulation of pro-survival exosomes by the drug ticagrelor on human cardiac-derived mesenchymal progenitor cells. Ticagrelor is an inhibitor of P2Y12 receptors, and inhibitors of this receptor have been widely used in the clinic for cardioprotection. This drug acts by increasing exosome levels and this leads to the promotion of mitosis in these cells. Whilst this drug is taken orally, there is also the potential that this could be delivered directly to the site of cardiac injury via a biomaterial drug carrier, providing the ability for a controlled release. In other cases, exosomes can be associated with negative effects on the heart, for example in the promotion of cardiac fibrosis. Statins are a drug type that are already widely used in the clinic in the prevention of heart disease through the lowering of cholesterol. The mechanism by which simvastatin protects against cardiac fibrosis was researched by Kuo et al. (286). They found that it regulated the release of exosomes from cardiomyocytes and reduced the effect of cardiac fibrosis induced by angiotensin II. Statins are another orally taken drug, however again this could be delivered via a biomaterial directly to the site of interest.

## Conclusion and Future Outlook

This review establishes clearly the huge potential of natural biomaterials in cardiac tissue engineering. Table 1 summarizes the advantages and disadvantages of these materials in the context of cardiac tissue engineering.

**Table 1:** Advantages and disadvantages of natural materials used for cardiac tissue engineering.

**Material**	**Advantages**	**Disadvantages**
EHT	• Can be easily shaped or cast to the complex geometry of the myocardium, and so can provide efficient bonding to the native tissue • Good electrical coupling is possible • Can be generated easily with minimal variation • Have similar characteristics to heart tissue, meaning that they are suitable for drug toxicology • CRISPR/Cas9 can be used in conjunction with pluripotent stem cells and EHTs to generate tissues with patient specific diseases • Can be fused together to create relatively large constructs	• A true adult cardiomyocyte phenotype has not been reproduced • Larger EHTs with sufficient cells for clinical relevance have not yet been produced • As of yet EHT viability is not maintained as vascularization is unable to reach the core of the grafts • A fibrotic interface is often seen between the myocardium and EHT and this can reduce the chance of definitive electrical coupling
Collagen	• It is inherently biocompatible, superior to that of many other natural polymers • It is inherently bioactive due to the presence of appropriate binding ligands for cardiac cell attachment • It has modifiable biodegradability • It has low antigenicity • Collagen scaffolds are versatile, with many relevant physical, chemical, mechanical, and morphological properties being tailorable to achieve specific functions • Collagen can be extracted in large quantity from a wide range of tissue sources at high purity, and at relatively low cost • It has an abundance of potential ligand sites to promote cellular activity during myocardial tissue regeneration • Collagen, in particular fibrillar type I, is the main constituent of the ECM of many hard and soft tissues • It supports myocyte alignment and contributes to matrix resistance to deformation during the cardiac cycle, playing an important role in the maintenance of myocardium shape, thickness, and stiffness	• The low stiffness of gel-like systems and poor ability to create a spatial bio-mimetic environment somewhat limits its *in vivo* applications • There is difficulty in designing collagen scaffolds that have nonlinear elasticity similar to the heart muscle and therefore it is difficult to develop a scaffold which beats synchronously with the recipient heart • There is an unmet need for vascularization which is crucial for adequate mass transport, cell survival, electromechanical integration and functional efficiency of the transplanted cardiac patch
Alginate	• Alginates are natural polysaccharides that are considered to be biocompatible, biodegradable, non-toxic, and non-immunogenic • The scope of the applications of alginates in the field of biomedicine is broad, including cell transplantation, drug, and protein delivery, and wound healing • It has a non-thrombogenic nature • Can be directly and locally injected into the infarcted myocardium or via intracoronary injection and therefore it's use doesn't require open surgery	• Mammals lack the alginase enzyme, therefore alginate is a non-degradable material, however, the partial oxidation of alginate chains promotes degradation under physiological conditions • Alginate hydrogels have poor bioresorbability and low cell adhesiveness, which may lead to adverse tissue interaction and poor wound-healing properties
PHAs	• Many polymers in the PHA family are highly flexible elastomers which make them ideal for soft tissue engineering • PHA derived tissue engineering scaffolds have the potential to maintain their structural integrity over a longer period due to surface degradation vs. bulk degradation observed in PLA and PLGA • They are highly biocompatible and bioresorbable • They have diverse mechanical properties • PHAs can be used for different aspects of cardiac tissue engineering such as patches, and valves • PHA based sutures are FDA approved • Other commercial products include mesh constructs for ventral and inguinal hernia repair; patches for tendon and ligament repair; mesh constructs for face and breast lifts • Can be processed to make a diverse range of materials, including 3D printed bespoke structures, electrospun (solution and melt) fiber sheets, gyrospun fiber sheets, porous 3D scaffolds, melt extruded and dip molded tubular structures, solvent cast films, hydrogels, microspheres, and nanospheres • PHAs are sustainable polymers produced using fermentation and do not need to be isolated from animal/human tissue	• The medical grade PHA production method is mostly quite expensive and not many commercial sources are available • Often, different PHAs require blending together in order to produce a material with suitable mechanical properties for cardiac applications • Some PHAs are susceptible to thermal degradation
Silk	• A variety of silk-based biomaterials have been approved by the FDA • Good adherence to native cardiac tissue • Cause little to no immunological response • Silk-based biomaterials have diverse tuneability • Its high elasticity makes silk a good biomaterial for cardiac applications as it has the mechanical properties to cope with the constant contraction and relaxation of the muscle • It has been shown to have good cell adhesion • Can be used to make a diverse range of structures, including fibers, foams, hydrogels, nanoparticles, films, and 3D printed structures • It is bioresorbable	• Silk usually has to be combined with other materials to make it suitable for cardiac applications • The natural production of silk by spiders leads to batch-to-batch variability due to different species and even within individual spiders
Chitin/chitosan	• They are biocompatible (287) • Can be processed into films, membranes (288), hydrogels, fibers, scaffolds, and sponges (289) • Chitin and chitosan gels can be used for drug delivery (290) • Chitin has an adhesive nature (289) which can be useful in applications such as myocardial patches • Chitin also has bactericidal and antifungal characteristics, which can reduce the risk of infection if used in an application that requires implantation (289)	• Chitin has a rigid crystalline structure, making it difficult to dissolve in common solvents (288) • Chitin and chitosan are derived from individual organisms (e.g., crustaceans, insects, fungi) (287) leading to batch-to-batch variability
Decellularized heart	• It is biocompatible as it is derived from animal or human donors • Can be used to make both myocardial patches and cardiac valve replacements (291) • This has a pre-existing structure; therefore, this material requires less processing	• Decellularized heart can't be processed into as many different forms as other natural materials • It cannot be degraded after implantation • If any cells remain after decellularization of a xeno- or homograft, this can elicit an immunogenic response once implanted (291)
Omentum	• Part of a patient's own omentum can be removed by a minimally invasive procedure (292) • It is biocompatible as it is usually taken from the patient being treated • Omentum-based hydrogels can be made and used to encapsulate cells (293) • Omentum can be made into a myocardial patch (294)	• Where used to make an implanted myocardial patch, two surgeries are required—one to harvest the omentum and one to implant the patch. Surgery comes with risks, especially for a patient with a heart condition

Biocompatibility is the main property that brings these biomaterials to the forefront of cardiac tissue engineering. In addition, the mechanical properties and the rate of degradation are two other crucial properties that have been investigated and found to be suitable for cardiac applications. Among these biomaterials, the naturally occurring matrices, fibrinogen, collagen, alginate, and silk result in hydrogels which are soft materials, highly suitable for cardiac repair. A small number of clinical trials have been carried out, to date, using hydrogels derived from natural biomaterials. A summary of these studies and the main results are outlined in Table 2.

**Table 2:** Clinical trials using natural biomaterials in cardiac repair.

**Study**	**Description**	**References**
Intracoronary delivery of engineered alginate implants—IK-5001 bioabsorbable cardiac matrix (BCM) (Bellerophon LLC)Clinical trial unique identifier: NCT01226563	• Testing safety and feasibility of strategy in patients recovering from an extensive MI • 27 patients with moderate-to-large ST-segment-elevation MI (STEMI) enrolled after successful revascularization • Within 7 days of MI, a 2 mm alginate implant was delivered by injection through the coronary artery related to the infarct • Implant injection didn't impair coronary flow or myocardial perfusion, shown by coronary angiography 3 min after injection • Implant did not cause any further myocardial injury • Assessment by 12-lead echocardiograms, 24 h Holter monitoring, blood tests, and heart failure questionnaires were carried out at 30, 90, and 180 days post-treatment • A 6-month follow-up with these tests showed that the implant was tolerated and caused no serious arrythmias, blood test abnormalities, other adverse effects, or death • Left ventricular preservation and ejection fracture was shown to be preserved compared to previous reports • Promising results led to a further study with IK-5001	(295)
IK-5001 multicenter, international, randomized, double-blind, controlled trialClinical trial unique identifier: NCT01226563	• Comparing the bioabsorbable cardiac matrix (BCM) with saline control to assess LV dilation and adverse clinical events within 6-months • 303 patients with large infarct areas after percutaneous coronary intervention (PCI) of a STEMI were enrolled • Randomized into groups and 201 given BCM and 101 given saline injection into the artery related to the infarct between 2 and 5 days after PCI • A 6-month follow up showed there was no significant difference in left ventricular end-diastolic volume index between the groups, with 14.1 ± 28.9 mL/m^2^ in the BCM group compared to 11.7 ± 26.9 mL/m^2^ in the saline group • No significant difference in Kansas City Cardiomyopathy Questionnaire score, New York Heart Association functional class, and 6-min walk time • Primary safety outcomes (cardiovascular death, further MI, stent thrombosis, target-vessel revascularization, significant arrhythmia, myocardial rupture) were similar between the two groups with 11.6% for BCM and 9.1% for saline, *p* = 0.37 • Concluded that BCM did not reduce left ventricular remodeling or adverse cardiac events after 6-months.	(296)
Intramyocardial injection of alginate hydrogel—Algisyl-LVR^TM^ (LoneStar Heart Inc.)Clinical trial unique identifier: NCT00847964	• Testing safety and feasibility in patients with dilated cardiomyopathy • 11 patients with symptomatic heart failure were enrolled in the study, but only 3 were reported • Injection of material into left ventricular wall during scheduled coronary artery bypass graft surgery (CABG) • A 3-month follow-up of the three patients showed a substantial decrease in end-systolic and end-diastolic volume • The patients also showed an increase in ejection fraction from 32 ± 8% to 47 ± 18%, and a 35% decrease in myofiber stress • Promising results, however very small number of patients is a limitation, and the simultaneous CABG procedure may have an unclear contribution to the results. The results however do show a greater change and more rapid improvement than reported after CABG treatment alone	(297)
Algisyl-LVR^TM^ international, multi-center, prospective, randomized, controlled trial (AUGMENT-HF)Clinical trial unique identifier: NCT01311791	• A trial to evaluate the safety and benefits of an alginate hydrogel for left ventricular modification • 78 enrolled patients with advanced chronic heart failure were randomized and 40 treated with alginate hydrogel injection directly into the left ventricle muscle in combination with the standard medical therapy, and 38 treated with the standard medical therapy alone • 35 patients who were treated with the alginate hydrogel had no device-related complications, 3 patients died within 30 days of surgery (8.6%) • At a 6-month follow-up the alginate hydrogel treatment showed an improvement in peak VO2 compared to the control, where *p* = 0.014 • The 6-min walk time and New York Heart Association functional class was also more improved in patients who underwent alginate hydrogel treatment compared to the control group • 58 of the initial 78 patients with heart failure completed 12-months of follow-up. There were nine deaths in the alginate hydrogel treatment group and four deaths in the control group • At the 12-month follow-up, alginate hydrogel was associated with improved peak VO_2_ compared to the control, where *p* < 0.001 • Statistically significant improvements in the 6-minute walk time, New York Heart Association functional class, and VO2 at anaerobic threshold were reported • This trial showed that the addition of the alginate hydrogel was more effective in improving patients' symptoms and exercise capacity compared to the standard medical treatment alone	(184, 298)
A Phase I, Open-label Study of the Effects of Percutaneous Administration of an Extracellular Matrix Hydrogel, VentriGel, Following Myocardial InfarctionClinical trial unique identifier: NCT02305602	• A trial to evaluate the safety and feasibility, and effects of VentriGel, an extracellular matrix hydrogel, delivered via trans-endocardial injection in post-MI patients • 15 enrolled patients who had had a first STEMI and treated with PCI in the last 3 years, with evidence of left ventricular dysfunction and remodeling • Approximately half of the enrolled patients were treated <12 months after MI and the other half more than 12 months after MI • VentriGel was well-tolerated with no deaths or patient dropouts from the trial • One patient suffered two cardiac events—cardiogenic shock and complete heart block—and one patient developed an intracardiac thrombus. These were reported as possibly due to the procedure, and no other adverse events due to either the VentriGel or the injection procedure were reported • The 6-min walk time was assessed at 3 and 6-month follow-ups, and VentriGel treatment was found to significantly increase the maximum distance walked at *p* = 0.004 • New York Heart Association functional class significantly decreased, *p* = 0.041, at 1, 3, and 6-month follow-ups, as with the heart failure questionnaire which significantly decreased, *p* = 0.045, at 1-month and non-significantly decreased at 3 and 6-months • MRI to evaluate cardiac function at 6-month follow-ups of 14 of the patients showed maintained or decreased left ventricular end-diastolic or end-systolic volume in comparison to baseline at the final follow-up, with this occurring predominantly in patients over 12 months post-MI over those <12 months post-MI • No significant changes were recorded in the ejection fraction or infarct scar size • This trial supports the safety and feasibility of VentriGel in post-MI patients, and improvements in left ventricular remodeling were observed • This first study using an injectable ECM hydrogel could lead to further randomized, controlled, larger clinical trials	(299)

These natural biomaterials have also been processed to obtain other types of 3D structures with tailored porosity, in order to incorporate an additional level of controlled microstructure, hence better mimicking normal tissue structure, including cardiac tissue. The other type of natural material discussed in this review are the ones that are produced using bacterial fermentation, i.e., PHAs. These have the advantage of being highly processable using a range of techniques and have varied mechanical properties, which can be tuned toward bespoke patient specific requirements. Considerations of promoting vascularity within the cardiac patches have been addressed in a number of ways, often by addition of vasculogenic factors, but production of large perfusable vessels is still a challenge. More research is needed toward the identification of methods to promote functional coupling between the graft and host cardiomyocytes, so as to prevent the arrhythmic effects that can be produced by bulk injection of cells. To achieve scaffolds with properties as close as possible to natural cardiac tissue, multi-material structures produced via 3D printing techniques with structures bespoke to patients, promise exciting advances in cardiac tissue engineering for the near future.

## Author Contributions

QM, AF, DG, ND, OH-C, RJ, and TO, all contributed equally and were involved in collecting the information for their section of the review and writing it. In addition QM and AF also contributed toward the collation of the final manuscript and the collation and formatting of the references. SH and IR are the senior authors and contributed by supervision and editing of the whole manuscript. MS, SB, RC, and SS are the senior authors and contributed by supervision and editing of their section of the manuscript. All authors contributed to the article and approved the submitted version.

## Conflict of Interest

The authors declare that the research was conducted in the absence of any commercial or financial relationships that could be construed as a potential conflict of interest.
